# IL-7R licenses a population of epigenetically poised memory CD8^+^ T cells with superior antitumor efficacy that are critical for melanoma memory

**DOI:** 10.1073/pnas.2304319120

**Published:** 2023-07-17

**Authors:** Goran Micevic, Andrew Daniels, Karine Flem-Karlsen, Koonam Park, Ronan Talty, Meaghan McGeary, Haris Mirza, Holly N. Blackburn, Esen Sefik, Julie F. Cheung, Noah I. Hornick, Lilach Aizenbud, Nikhil S. Joshi, Harriet Kluger, Akiko Iwasaki, Marcus W. Bosenberg, Richard A. Flavell

**Affiliations:** ^a^Department of Immunobiology, Yale School of Medicine, New Haven, CT 06520; ^b^Department of Dermatology, Yale School of Medicine, New Haven, CT 06520; ^c^Department of Pathology, Yale School of Medicine, New Haven, CT 06520; ^d^Department of Surgery, Yale School of Medicine, New Haven, CT 06520; ^e^Yale Cancer Center, Yale School of Medicine, New Haven, CT 06520; ^f^Department of Medicine (Medical Oncology), Yale School of Medicine, New Haven, CT 06520; ^g^Yale Stem Cell Center, Yale School of Medicine, New Haven, CT 06520; ^h^HHMI, Chevy Chase, MD 20815; ^i^Yale Center for Immuno-Oncology, Yale School of Medicine, New Haven, CT 06520

**Keywords:** melanoma, immunology, immunotherapy

## Abstract

Recurrence of advanced melanoma after therapy is a major risk factor for reduced survival, and treatment options are limited. Antitumor immune memory plays a critical role in preventing melanoma recurrence and memory T cells could be a potent cell-based therapy, but the identity, and functional properties of the required immune cells are incompletely understood. Here, we show that an IL-7R^hi^ tumor-specific CD8^+^ population is critical for antitumor memory and can be epigenetically augmented to drive powerful antitumor immune responses. Using a model of functional antimelanoma memory, we found that high IL-7R expression selectively marks a CD8^+^ population in lymphoid organs that plays critical roles in maintaining tumor remission after immunotherapy or surgical resection. This population has intrinsic cytotoxic activity, lacks markers of exhaustion and has superior antitumor efficacy. IL-7Rhi cells have a functionally poised epigenetic landscape regulated by DNA methylation, which can be augmented by hypomethylating agents to confer improved survival and complete melanoma clearance in naive mice. Importantly, greater than 95% of tumor-specific T cells in draining lymph nodes after therapy express high levels of IL-7R. This overlap between IL-7R^hi^ and antigen-specific T cells allows for enrichment of a potent functional CD8^+^ population without determining antigen-specificity, which we demonstrate in a melanoma model without a known antigen. We identify that IL-7R expression in human melanoma is an independent prognostic factor of improved survival. These findings advance our basic understanding of antitumor memory and suggest a cell-based therapy using high IL-7R expression to enrich for a lymph node population with superior antitumor activity that can be augmented by hypomethylating agents.

## Significance

Treatment options for patients with recurrent melanoma are limited and understanding antitumor memory is critical to prevent recurrence and develop improved therapies. Previous studies found that adoptively transferring antigen-specific T cells with memory traits provides improved clinical benefit, suggesting that antitumor memory T cells could be the ideal candidate for cell-based therapies. However, this population and its markers remain elusive. We analyzed tumor-specific T cells using single-cell approaches in a model of antimelanoma memory. Our results identify a CD8^+^ population selectively marked by IL-7R expression that drives antitumor memory and can be used as a potent therapy for melanoma. The antitumor function of this population can be epigenetically augmented to develop powerful adoptive cell therapies that could improve melanoma survival.

Despite significantly improved outcomes with anti-CTLA-4 and anti-PD-1/PD-L1 inhibitors, approximately half of patients with advanced melanoma will not achieve a durable response and face a high risk of recurrence and death (1). Treatment options for patients with recurrent melanoma are limited, and there is a major unmet need to both understand the determinants of a durable response and develop therapies for recurrent melanoma. Cell-based immunotherapies using adoptively transferred T cells (ACT) hold great potential for patients with advanced melanoma recurrence (2–4). For instance, patients with advanced melanoma refractory to anti-PD-1 therapy who receive adoptive transfer of tumor infiltrating lymphocytes (TILs) have better survival compared with anti-CTLA-4 blockade (1). Cases of remission have been reported in subgroups of patients; however, significant challenges limiting the benefit of ACT remain (5). These challenges include: 1) enriching for tumor-specific T cells, 2) selecting the optimal T cell population with intrinsic characteristics suitable to mediate effective antitumor responses, and 3) overcoming contextual signals in the tumor microenvironment (TME) and chronic TCR stimulation that drive T cell dysfunction/exhaustion (6).

ACT effectiveness correlates with the ability to transfer tumor-specific T cells which can recognize and kill cancer cells (7–9), however such T cells make up only a small fraction of TILs. TILs also include suppressive T regulatory cells (10) and CD39-expressing T cells that limit antitumor immunity (11). Additionally, TILs lack the memory/stemness properties (12, 13) that correlate with clinical benefit upon transfer (14, 15). Tumor-specific T cells from the TME are largely terminally differentiated (16) and express markers of exhaustion, such as PD-1, LAG-3, and TIM-3 (17, 18), limiting their functional antitumor activity. T cell exhaustion also occurs during chronic viral infections, where epigenetic scarring (19, 20) limits the effectiveness of viral-specific lymphocytes similar to what is observed in the TME (21–23). Despite persistent antigen encounters promoting exhaustion, long-lived memory CD8^+^ populations with intrinsic stemness features can sustain a T cell response and immunity to chronic infection (24–26). However, the identity of an analogous CD8^+^ T cell population that sustains antitumor memory is currently elusive. Identifying and characterizing an antitumor memory population is important, not only because it would advance our understanding of antitumor immunity, but would be a potential source of tumor-specific cells with memory/stemness properties, which have shown promising antitumor efficacy in preclinical and small clinical studies (12, 13, 27, 28).

Herein, we use an experimental model of antimelanoma memory to identify and characterize an endogenous population of IL-7R^hi^ CD8^+^ T cells with superior antitumor activity that plays critical roles in antitumor memory. This tumor-specific *IL-7R^hi^ CD8^+^* population resides in lymphoid organs, has memory and intrinsic cytotoxic features, and lacks transcriptional and epigenetic markers of exhaustion. The distinct epigenetic landscape of this *IL-7R^hi^ CD8^+^* population plays central roles in its antitumor function and can be augmented by hypomethylating agents. We find that IL-7R signaling is essential for this cell phenotype and selecting for high IL-7R expression can be used to enrich for cells with superior antitumor function even in tumors without known antigens. Integrating our findings with studies from human melanoma, we find that the *IL-7R^hi^* CD8^+^ signature is an independent prognostic factor of survival. These findings extend our fundamental understanding of antitumor memory and suggest that adoptive transfer of *IL-7R^hi^* memory populations in combination with epigenetic therapies could be used to develop cell-based therapies for melanoma.

## Results

### CD8^+^ T Cells Are Necessary for Functional Antitumor Memory.

To identify the T cell populations necessary for a successful antitumor memory response, we used the YUMM1.7 (Braf^V600E^Cdkn2a^−/−^) melanoma model (29), engineered to express the LCMV gp33-41 and gp66-77 antigens as well as a fluorescent label (YUMM-GFP33/66) or chicken ovalbumin (YUMM-OVA) (30) (*SI Appendix*, Figs. S1*A* and S6). These dominant antigen models uniformly form tumors in immune competent mice but are robustly immunogenic (*SI Appendix*, Fig. S1*B*) and recapitulate key properties of human melanoma in the context of immunotherapy, including response to PD-1 and CTLA-4 blockade (*SI Appendix*, Fig. S1*C*), presence of progenitor-exhausted and exhausted T cell populations (*SI Appendix*, Fig. S1*D*) and response to adoptive cell transfer (*SI Appendix*, Fig. S1*E*) while facilitating interrogation of antigen-specific T cells.

**Fig. 1.**
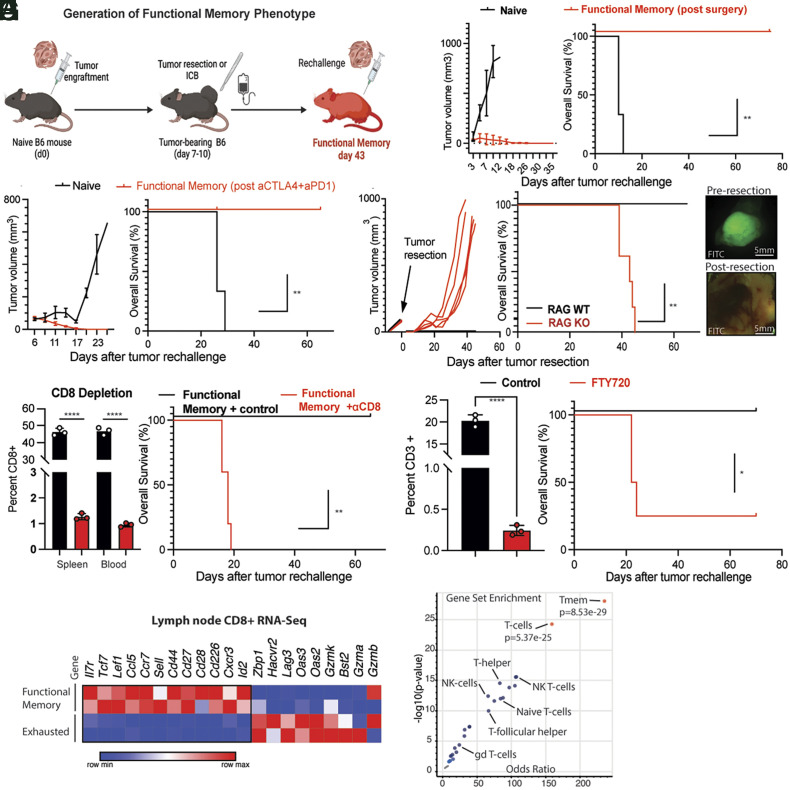
CD8^+^ T cells are necessary for functional antitumor memory. (*A*) Diagram summarizing generation of functional memory mice. Wild-type C57BL/6 mice were injected with 1 to 2 × 10^6^ YUMM-OVA or YUMM-GFP33/66 cells on day zero. Mice were treated with either surgical resection (day 10) or immunotherapy (anti-CTLA-4 and anti-PD1, days 8 to 18). Tumor rechallenge with 2 × 10^6^ YUMM-OVA or YUMM-GFP33/66 melanoma cells was done on day 43. (*B*, *Left*) Tumor growth upon rechallenge of functional memory mice (red) generated by tumor resection (*B*, *Left*) or control naive mice (black) with 3 × 10^6^ YUMM-GFP33/66 melanoma cells. (*B*, *Right*) Kaplan–Meier curve comparing survival of age-matched functional memory or control (naive) mice upon tumor rechallenge. (*C*, *Left*) Tumor growth upon rechallenge of functional memory mice (red) generated by complete response to immunotherapy or control naive mice (black) with 3 × 10^6^ YUMM-OVA or YUMM-GFP33/66 melanoma cells. (*C*, *Right*) Kaplan–Meier curve comparing survival of age-matched functional memory or control (naive) mice upon tumor rechallenge. (*D*, *Left*) Tumor growth upon resection of RAG WT (black) and RAG KO (red) mice. (Middle) Kaplan–Meier curve comparing survival of age-matched Rag KO (red line) and WT mice (black line) after resection of YUMM-OVA or YUMM-GFP33/66 tumors. RAG KO mice fail to develop functional memory and tumors recur at the site of resection. (*D*, *Right*) fluorescent images taken preresection (*D*, *Top*) and postresection (*D*, *Bottom*) of GFP-expressing YUMMER1.7GPF tumors under fluorescent dissecting microscope. Normal autofluorescence seen in lower image but no sign of tumor cells. (*E*, *Left*) Comparison of CD8^+^ percent in spleen and circulation of CD8 depleted (red) and sham-treated functional memory mice (black).(*E, Right*) Kaplan–Meier curve comparing survival of functional memory mice treated with isotype control (control, black line) or CD8 depletion (red line). CD8 depletion was done for 3 wk beginning on day 40, 3 d prior to tumor rechallenge with YUMM-OVA or YUMM-GFP33/66 melanoma cells. (*F*, *Left*) Comparison of CD3^+^ percent in circulation of FTY-720-treated (red) and sham-treated functional memory mice (black). (*F*, *Right*) Kaplan–Meier curve comparing survival of the control (black) and FTY720 (red)-treated groups. Functional memory mice were treated with FTY720 (red line) or phosphate buffered saline (black line) beginning on day forty, 3 d prior to YUMM-OVA or YUMM-GFP33/66 rechallenge. FTY720 administration leads to failure of functional memory. (*G*) Heatmap summarizing scaled expression values of a subset of variable genes between CD8+ T cells from lymph nodes of functional memory and exhausted mice (RNA-Seq data). (*H*) Volcano plot of top gene-sets enriched in CD8+ T–cells from tumor-draining lymph nodes of functional memory mice. Tmem (*P* = 8.53 × 10^−29^) and T–cells (5.3 × 10^−25^) are the top overrepresented cell types/gene signatures (red). Student’s *t* test, ANOVA or Log-rank test; *P < 0.05, **P < 0.01, ***P < 0.001. Bar graphs shown as mean +/− SEM, based on three biological replicates.

Using these reagents, we developed a model of functional antitumor memory (Fig. 1*A*) which exhibits 100% tumor rejection upon rechallenge. To acquire functional memory, wild-type C57BL/6 (naive) mice are initially grafted (challenged) with YUMM-GFP33/66 or YUMM-OVA tumor cells before the tumor is cleared by surgical resection or combined anti-PD-1/anti-CTLA-4 blockade. Four to six wk after tumor clearance by immunotherapy or surgery, mice are rechallenged with 1 to 10 × 10^6^ tumor cells, and a robust memory response characterized by rapid tumor rejection is seen in all tested mice (Fig. 1 *B* and *C*). We validated the functional memory phenotype with a widely used syngeneic colon cancer model, MC38, as well as a nondominant antigen-expressing model, YUMMER1.7 (*SI Appendix*, Fig. S1*F*) (31). Importantly, this memory response is tumor specific, and tumor rejection does not occur upon rechallenge with a different tumor e.g., YUMM-GFP33/66-derived functional memory mice fail to reject a rechallenge with MC38. (*SI Appendix*, Fig. S1*G*). Importantly, functional memory cannot be generated in immunodeficient RAG^−/−^ mice, due to universal spontaneous tumor recurrence after resection (Fig. 1*D*). Recurrence in RAG^−/−^ mice occurs despite confirming surgically complete resections using a fluorophore-labeled tumor line (YUMMER1.7GFP) under a microscope (Fig. 1*D*). This observation suggests that mature lymphocytes are necessary to prevent tumor recurrence even after resection and to lay the foundations for memory.

To further investigate which immune cells are required for functional memory, we depleted CD8^+^ T cells (Fig. 1*E*). The functional memory phenotype was abolished by depletion, suggesting that CD8^+^ T cells are necessary for functional antitumor memory.

Notably, we detected a significant expansion of dominant antigen-specific CD8^+^ T cells in both the tumor-draining lymph node (tdLN) as well as the spleens of mice after YUMM-GFP33/66 challenge (*SI Appendix*, Fig. S2*A*). Additionally, a continued proportional expansion of these CD8^+^ T cells 6 wk after tumor resection is seen only in the tdLN (*SI Appendix*, Fig. S1*H*). These observations together led us to hypothesize that influx of T cell populations from lymphoid organs, and particularly the tdLN, is necessary for the antitumor memory response. To test this hypothesis, we treated functional memory mice with the sphingosine 1-phosphate receptor-1 (S1PR1) inhibitor FTY-720 (32), which blocks lymphocyte egress from lymphoid organs. Majority of the memory mice treated with FTY-720 failed to mount an effective antitumor memory response. Thus, blocking lymphocyte egress from lymphoid organs significantly blunted the memory phenotype (Fig. 1*F*), and suggests that the CD8^+^ T cell population(s) necessary for functional antitumor memory reside in lymphoid organs.

We next isolated CD8^+^ T cells from lymphoid organs of functional memory mice and analyzed their transcriptome using bulk-RNA Seq. We used lymph nodes from mice with outgrowing tumors (exhausted) as control. We performed gene set enrichment analysis and found that CD8^+^ T cells in lymph nodes of functional memory mice had a T cell memory–like transcriptional profile (Fig. 1 *G* and *H*), including high expression of *Il7r*. IL-7R is the interleukin-7 receptor (also known as CD127), which is critical for T cell development, survival, and memory differentiation (33–36). Whether this high IL-7R expression was marking a tumor-specific CD8+ population became important to address.

### An IL-7R^hi^ CD8^+^ Population Plays a Critical Role in Antitumor Memory.

**Fig. 2.**
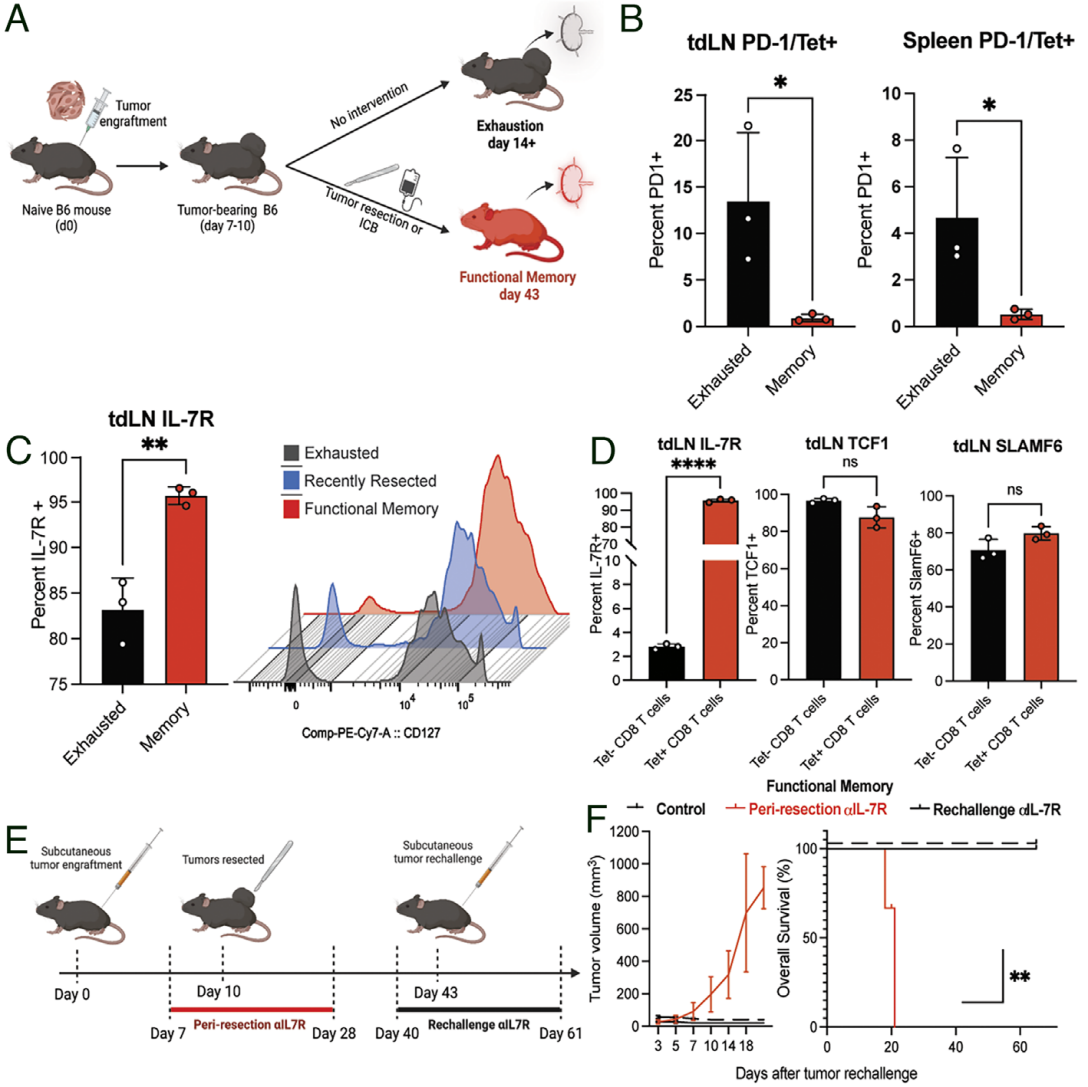
An IL-7R^hi^ CD8^+^ population plays a critical role in antitumor memory. (*A*) Diagram illustrating generation of exhausted and functional memory mice. Wild-type C57BL/6 mice were injected with 1 to 2 × 10^6^ YUMM-OVA or YUMM-GFP33/66 tumors on day zero. For functional memory generation, mice started receiving immunotherapy on day eight (five treatments of combined anti-CTLA4/anti-PD-1) or surgical resection on day ten. Functional memory mice were used for experiments 2 wk after tumor clearance. For exhausted mice, no therapy was administered. Exhausted mice were used for experiments 2 wk after initial tumor injection. (*B*) Percent PD-1 expression in exhausted (black) versus functional memory (red) tumor-specific (Tet+) CD8^+^ T cells in tumor-draining lymph nodes (tdLN) and spleen. (*C*) Percent IL-7R positivity of tumor-specific CD8^+^ T cells (*C*, *Left*) in exhausted (black) versus functional memory (red) tdLN and histogram overlay (*C*, *Right*) of IL-7R expression in CD8^+^ T cells of exhausted (gray), recently resected (blue) and functional memory (red) mice. (*D*) Percent positivity of key memory and stemness markers in antigen-specific (tetramer positive, red) versus polyclonal (tetramer negative, black) CD8^+^ T cells in the tumor-draining lymph node of functional memory mice. (*E*) Diagram summarizing IL-7R blocking antibody administration. Mice were treated for 21 d, starting either 3 d prior to resection (periresection, red bar days 7 to 28) or 3 d prior to rechallenge (rechallenge, black bar days 40 to 61). See also Fig. 2*F*. (*F*) Tumor volume (*F*, *Left*) and Kaplan–Meier curves (*F*, *Right*) showing tumor growth after rechallenge of functional memory mice, which received no additional therapy (solid black line), IL-7R blockade at time of rechallenge (dashed line), or periresection IL-7R blockade (red line). Student’s *t* test, ANOVA or Log-rank test; *P < 0.05, **P < 0.01, ***P < 0.001. Bar graphs shown as mean +/− SEM, based on three biological replicates.

IL-7R can mark multiple T cell states (37). IL-7R is expressed by naive T cells, is rapidly lost upon T cell activation and is absent from most effector T cell populations but reexpressed by several types of memory CD8^+^ T cells (38, 39). We next sought to investigate whether IL-7R was expressed on tumor antigen-specific T cells. We designed a flow-cytometry panel equipped to detect antigen-specific T-cells (*SI Appendix*, Fig. S2*A*), canonical memory markers (*SI Appendix*, Fig. S2*C*) and markers of T cell exhaustion (*SI Appendix*, Fig. S2*B*). We used this panel to interrogate the tumor-specific and polyclonal CD8^+^ T cells in the tdLN and spleen of functional memory and exhausted mice (Fig. 2*A*). Unsurprisingly, tumor-specific (tetramer positive) CD8^+^ T cells from exhausted conditions (outgrowing tumors) showed increased expression of exhaustion/activation marker PD-1 when compared with functional memory (Fig. 2*B*). Conversely, functional memory mice had a significant increase in the proportion of tumor-specific T cells marked by IL-7R compared with exhaustion (Fig. 2*C* and *SI Appendix*, Fig. S2*D*).

Strikingly, we found that IL-7R was highly expressed on the surface of tumor-specific CD8^+^ T cells (IL-7R^hi^) in functional memory, but not on tetramer-negative CD8^+^ T cells (IL-7R^lo^) in this setting, making it unique in this aspect from other canonical markers of memory that were evaluated (Fig. 2*D*). Only 3% of tetramer-negative CD8^+^ T cells were IL-7R^+^ (Fig. 2*D*). However, the population of IL-7R^+^ CD8^+^ T cells accounted for over 90% of tumor-specific CD8^+^ T cells (Fig. 2*D*) in lymph nodes in conditions associated with functional memory. The proportion of tumor-specific IL-7R^hi^ CD8^+^ T cells was highest in the tdLN and expanded over time from approximately 85% after initial tumor clearance to over 95% after 2 wk (*SI Appendix*, Fig. S2*D*). This population did not express exhaustion-associated markers PD-1 or TIM-3 (*SI Appendix*, Fig. S2*F*).

To determine if this expanding IL-7R^hi^ tumor-specific CD8^+^ population is functionally important for antitumor memory, we blocked IL-7R signaling both after initial tumor challenge (priming) or during functional memory rechallenge (Fig. 2*E*). Mice treated with IL-7R blocking antibody after priming, but not during rechallenge, failed to form functional antitumor memory and exhibited tumor formation and growth identical to naive mice upon rechallenge (Fig. 2*F*). Administration of IL-7R blocking antibody immediately prior to functional memory rechallenge had no effect on the effective memory phenotype (Fig. 2*F*). CD8^+^ T cell levels were unaffected at the time of rechallenge (*SI Appendix*, Fig. S2*E*). Mice receiving IL-7R blockade during priming (Fig. 2*F*) had a median survival of 21 d. The median survival in functional memory mice (control) was undefined, since all tumors were rejected. Thus, IL-7R blockade after priming prevented formation of functional antitumor memory. Taken together, these findings suggest that an IL-7R^hi^ tumor–specific CD8^+^ population in lymphoid organs is critical for antitumor memory.

### A Tumor-Specific IL-7R^hi^ CD8^+^ T Cell Population Has Central Memory-Like Features.

**Fig. 3.**
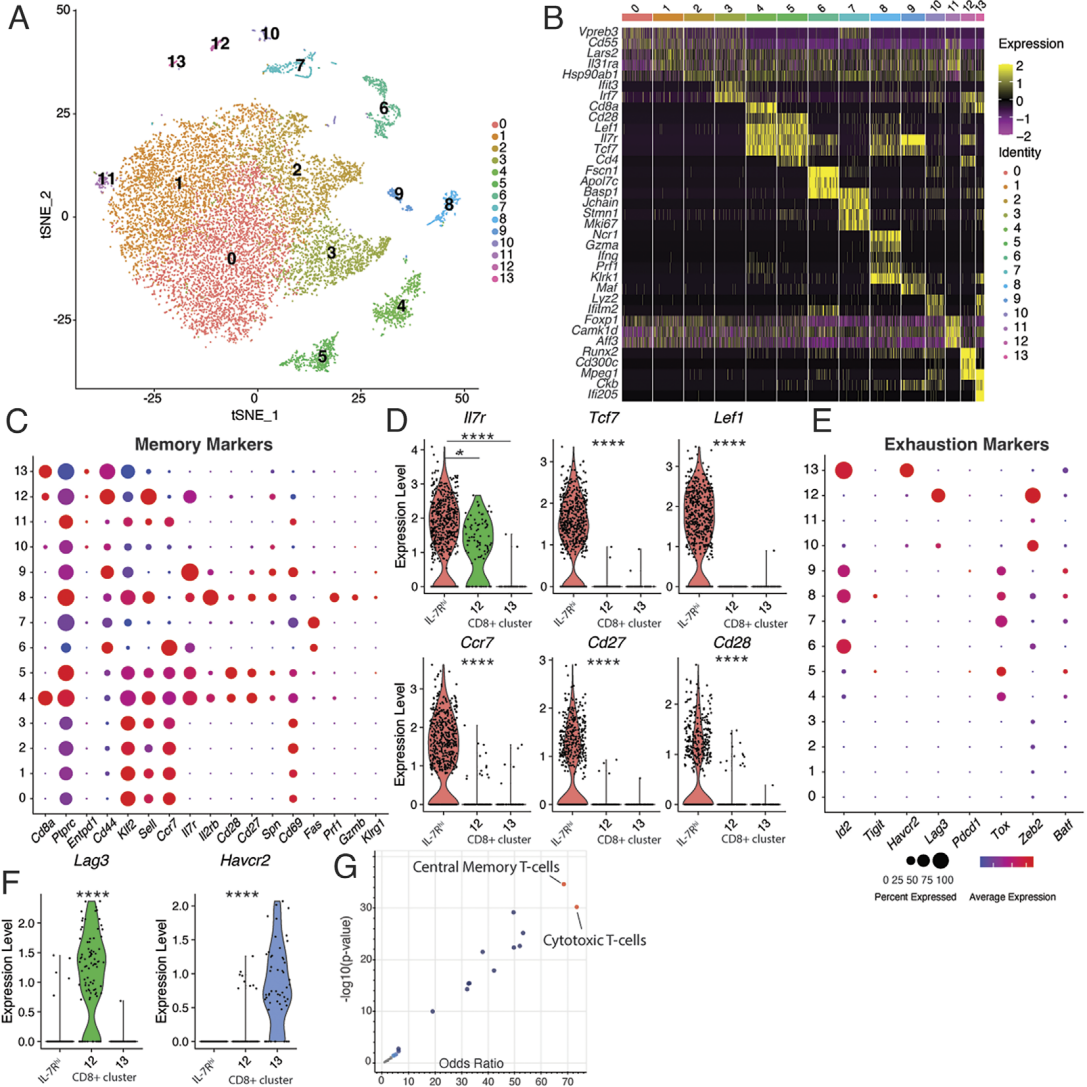
A tumor-specific IL-7R^hi^ CD8^+^ T cell population has central memory-like features. (*A*) tSNE (t-distributed stochastic neighbor embedding) plot of 13 distinct cell clusters from tumor-draining lymph nodes of functional memory mice (n = 13,963 cells). Cells are colored based on clusters identified by the Louvain algorithm. See also *SI Appendix*, Fig. S3 *C* and *D* for automated cell type identification and their connectedness. (*B*) Heatmap summarizing scaled expression values of marker genes for each individual cluster, as shown in *A*. See also *SI Appendix*, Fig. S3 *A* and *B*. (*C*) Dot plot showing relative mean expression level of markers of T cell memory–associated genes. (*D*) Violin plots of a subset of memory marker genes in CD8^+^ clusters 4 (IL-7R^hi^), 12 and 13. (*E*) Dot plot showing relative mean expression level of markers of exhaustion-associated genes. (*F*) Violin plots of a subset of exhaustion marker genes in CD8^+^ clusters 4 (IL-7R^hi^), 12 and 13. (*G*) Volcano plot of top gene-sets enriched among the tumor-specific IL-7R^hi^ CD8^+^ cluster. Central Memory T cells and Cytotoxic T cells are the top overrepresented gene sets (red). Student’s *t* test or ANOVA; *P < 0.05, **P < 0.01, ***P < 0.001. Bar graphs shown as mean +/− SEM, based on three biological replicates. Violin plots show the median (dashed line) and quartiles (dotted lines). Cytokines measured by ELISA using recombinant protein standard curve, based on three biological replicates.

To further characterize the IL-7R^hi^ CD8^+^ cell population important for antitumor memory, we used scRNA-seq to characterize the immune cell populations in tumor-draining lymph nodes from mice with functional memory, including both tumor-specific and nonspecific (polyclonal) populations. After dimensionality reduction, graph-based clustering and mapping gene expression in two dimensions via t-distributed stochastic neighbor embedding (tSNE), we identified 13 distinct cell clusters (Fig. 3*A*) from the tdLN of functional memory mice. Top marker genes were identified for each cluster (Fig. 3*B* and *SI Appendix*, Fig. S3 *A* and *B*). Clusters 0 to 3 were marked by canonical B cell markers, included naive and pre-B cells. Cluster 4 was marked by *Cd8a* expression, while cluster 5 consisted of CD4 cells. Both clusters 4 and 5 expressed high levels of *Il7r* (Fig. 3*C*). Overall, CD8 expression was found in clusters 4, 12, and 13 of the datasets, and those clusters were selected for further analysis (*SI Appendix*, Fig. S3*F*). The remaining clusters in the dataset included natural killer cells (cluster 8), γδ-T cells (cluster 9), neutrophils (cluster 6), and monocytes (cluster 10) (*SI Appendix*, Fig. S3*C*).

We characterized the clusters more closely with a panel of common markers of memory and exhaustion (Fig. 3 *C*–*F*). Cluster 4 showed high expression of Il7r, Ccr7 (C-C chemokine receptor type 7), Sell (also known as CD62L), Cd27 and Cd28. Ccr7 is expressed by naive, regulatory, and central memory T cells, and plays important roles in regulating T cell migration and homing to lymph nodes (40). As opposed to Ccr7, which mediates ‘hard stop’ signaling and transmigration into the lymph node, Sell is an L-selectin that mediates the initial rolling adhesion and deceleration. It is expressed on naive and central memory T cells and is generally absent from effector T cell populations (41). Tcf7 is a Wnt signaling target essential for T cell memory differentiation and self-renewal and is essential for formation of central memory T cells (42–44). Cluster 4 also exhibited high expression of *Cd28* (Fig. 3*D*), which is required for proliferation of stem-like cells (45, 46) as well as *Eomes*, a transcription factor with important roles in generation and persistence of memory CD8^+^ T cells. High levels of *Bcl-2*, which can eschew apoptotic death of lymphocytes and promotes long-term survival of memory populations (47–49) was notable in cluster 4 (*SI Appendix*, Fig. S3*E*). Entpd1 (CD39), which has recently been shown to play immunosuppressive functions in the TME (11) was not expressed by cluster 4 (Fig. 3*C*).

Markers of exhaustion, such as *Pdcd1*, *Havcr2*, *Lag3*, *Tigit,* and *Id2* were absent from cluster 4 (Fig. 3*E*). Clusters 12 and 13 were also CD8^+^; however, they did not express memory markers (Fig. 3*D*). In contrast, they expressed markers of exhaustion (Fig. 3*F*). We also profiled the TME from mice at day 14 after tumor grafting (5,192 cells). This condition represents an exhausted phenotype generated by grafting YUMM-GFP33/66 cells subcutaneously and not providing any therapy. A failed immune response and uniform tumor outgrowth occurs in this setting. In contrast to the functional memory signature (Fig. 3*E*), nearly all CD8^+^ cells in the exhausted TME expressed high levels of exhaustion markers *Pdcd1*, *Havcr2* and *Lag3* (*SI Appendix*, Fig. S3*G*).

Based on our findings, cluster 4 likely represents the IL-7R^hi^ CD8^+^ population that is critical for functional antitumor memory. It recapitulated the key features detected by flow cytometry, including high expression of IL-7R (Fig. 2*D*), absence of exhaustion markers (Fig. 2*B*) and tumor-specificity. Interestingly, gene set expression analysis of tumor-specific CD8+ T cells in cluster 4 showed enrichment of central-memory (T_CM_)-like and cytotoxic-like T cell phenotypes (Fig. 3*G*).

### Tumor-Specific IL-7R^hi^ Cells Constitutively Express Intrinsic Cytotoxicity Mediators, Have Superior Antitumor Function.

**Fig. 4.**
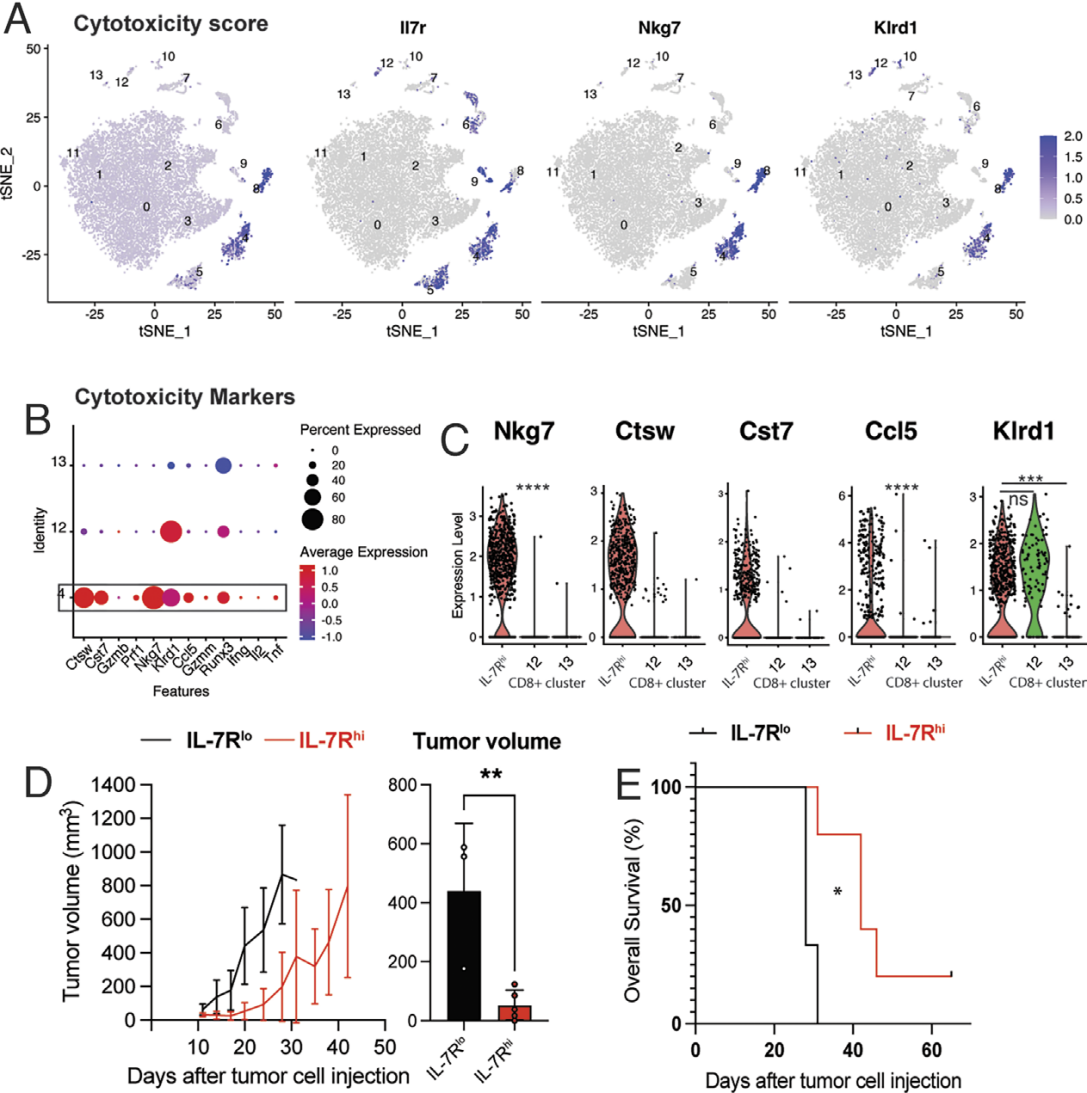
Tumor-specific IL-7R^hi^ cells constitutively express intrinsic cytotoxicity mediators, have superior antitumor function. (*A*) Single-cell transcription level and distribution of expression for indicated genes in the tSNE plot from Fig. 3*A*. Level of transcription displayed as gradient from gray (no expression) to purple (expression). Cytotoxicity score (*A*, *Left*) based on expression of Ctsw, Cst7, Gzmb, Prf1, Nkg7, Klrd1, Ccl5, Gzmm, Runx3, Ifng, Il2 and Tnf. (*B*) Dot plot showing relative mean expression level of markers of cytotoxicity across the CD8+ T cell clusters 4 (IL-7R^hi^), 12 and 13. (*C*) Violin plots of a subset of cytotoxicity marker genes in CD8^+^ clusters 4 (IL-7R^hi^), 12 and 13. (*D*, *Left*) Tumor volume curves for C57BL/6 mice grafted with melanoma cells after receiving an IL-7R^hi^ (red) or IL-7R^lo^ (black) population by adoptive transfer from tdLN of functional memory mice. (*D*, *Right*) Comparison of tumor volume at day 21 post tumor cell injection in the IL-7R^hi^ (red) and IL-7R^lo^ (black) recipient group. (*E*) Kaplan–Meier survival curve for tumor-bearing mice from indicated groups described in *D*. Student’s *t* test or ANOVA; *P < 0.05, **P < 0.01, ***P < 0.001. Bar graphs shown as mean +/– SEM, based on three biological replicates. Violin plots show the median (dashed line) and quartiles (dotted lines). Five mice were assigned to each experimental group.

We next sought to further investigate the potential cytotoxic properties of the IL-7R^hi^ CD8^+^ T cells in cluster 4. We used the composite expression of 12 cytotoxicity-associated genes to calculate an overall cytotoxicity score for all cell clusters. IL-7R^hi^ CD8^+^ T cells (cluster 4) and NK cells (cluster 8) had the highest cytotoxicity score (Fig. 4*A*). Tumor-specific IL-7R^hi^ CD8^+^ T cells (cluster 4) expressed intrinsic cytotoxic mediators including natural killer cell granule protein 7 (Nkg7), killer cell lectin receptor D1 (*Klrd1*), cathepsin W (Ctsw), cystatin F (Cst7), and *Ccl5* (Fig. 4*B*), suggesting a capacity for cytotoxicity. A high level of *Nkg7* expression was reported on antigen-specific CD8+ T cells, on cytotoxic lymphocytes (CTLs) infiltrating tumors in patients treated with immunotherapy (50) and regulates cytotoxic granule exocytosis in effector lymphocytes (51, 52). Ccl5 plays important roles in the onset of a proliferative burst in the setting of antigen reencounter (53–55). Ctsw and Cystatin F (Cst7) are membrane-associated cysteine protease whose expression is restricted to cytotoxic cells (56), are secreted during target cell killing (57) and regulate cytotoxicity in NK cells and effector-memory T cells (Tem) (58). Genes canonically associated with a CD8^+^ T cell effector signature (59), such as Gzmb, Prf1, *Ifng* were not highly expressed by IL-7R^hi^ CD8+ T cells (cluster 4) (Fig. 4*B*). The other CD8+ clusters, 12 and 13, had no appreciable or significantly lower expression of these cytotoxicity-associated genes compared with the IL-7R^hi^ CD8^+^ cluster (Fig. 4*C*).

Antigen-specific memory T cells are long-lived, respond rapidly to antigen rechallenge, and migrate efficiently to sites of rechallenge, making them particularly well for cell-based antitumor therapies (60). As noted previously, identifying a cell surface marker to indirectly enrich for tumor-specific T cells without the need to explicitly determine tumor antigens could increase the clinical benefit of ACT (61). As we have shown above, >95% of tumor-specific T cells expressed high levels of IL-7R (Fig. 2*D*). Additionally, based on the central memory-like and cytotoxic traits we found in IL-7R^hi^ CD8^+^ cells, we hypothesized they could be used for adoptive cell transfer without the need to identify tumor antigens, which is usually not feasible in clinical practice.

To test this possibility, we adoptively transferred IL-7R^hi^ and IL-7R^lo^ CD8^+^ cells from tdLN of functional memory mice into wild-type mice (Fig. 4*D*). We subsequently injected the recipient naive mice with tumor cells and monitored tumor growth and survival. Mice receiving IL-7R^lo^ CD8^+^ T cells showed similar tumor growth kinetics as negative controls (no ACT), with no significant difference in median overall survival between the IL-7R^lo^ and negative control group (Fig. 4*E*). By contrast, transfer of IL-7R^hi^ cells conferred a twofold decrease in tumor growth rate (Fig. 4*D*), significantly prolonged overall survival by 50%, and cured a subset of mice (Fig. 4*E*).

Collectively, these experiments suggest that IL-7R is a functional marker of a tumor-specific memory/cytotoxic CD8^+^ T cell population with superior antitumor function.

### IL-7R^hi^ CD8^+^ Cells Have a Distinct Poised Epigenetic Landscape Regulated by DNA Methylation That Imparts Superior Antitumor Function.

**Fig. 5.**
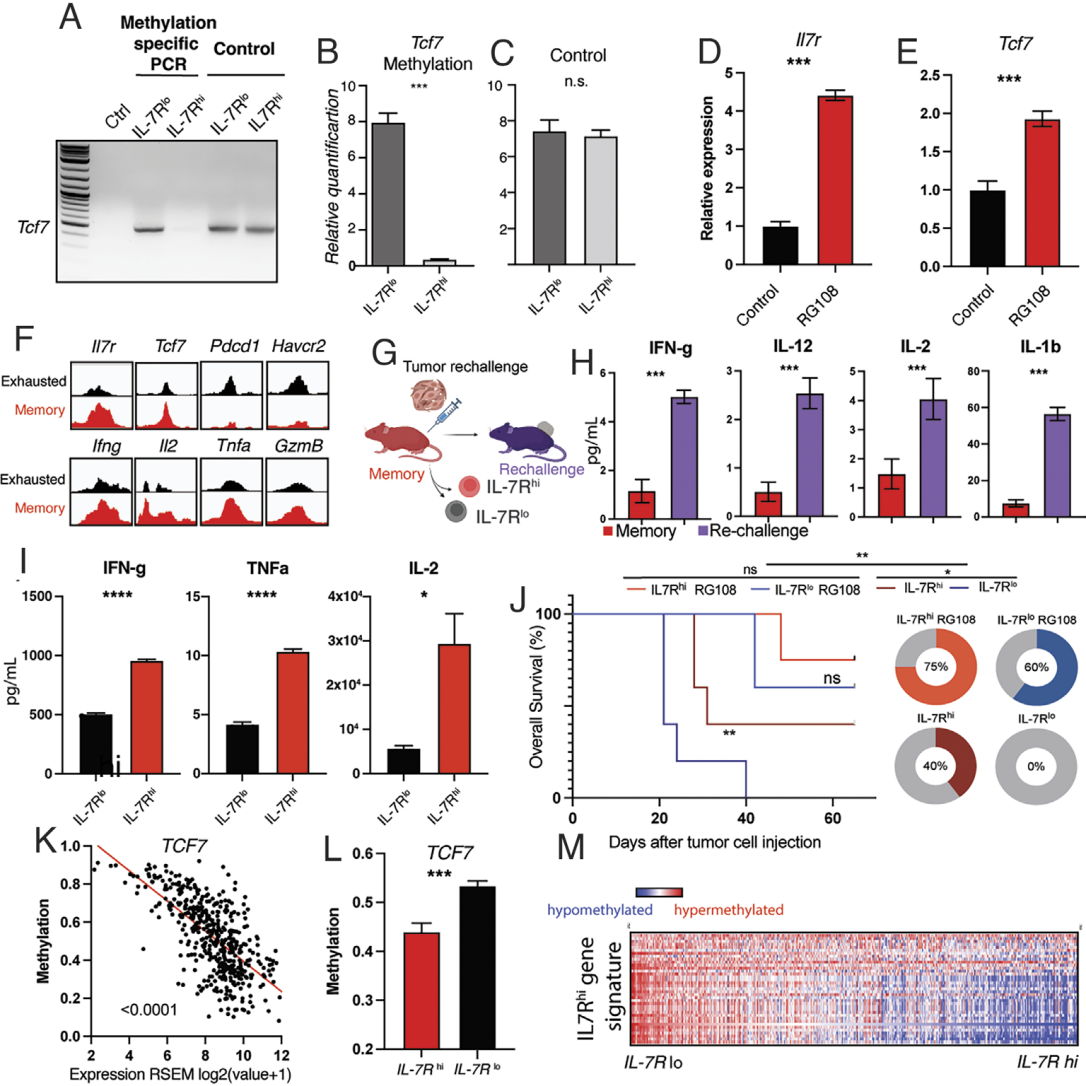
IL-7R^hi^ CD8^+^ cells have a distinct poised epigenetic landscape regulated by DNA methylation that imparts superior antitumor immunity. (*A*) Methylation-specific PCR targeting CpG loci in *Tcf7* promoter region in nonmethylated DNA standard (Ctrl, leftmost lane), IL-7R^lo^ and IL-7R^hi^ CD8^+^ T cells from tumor-draining lymph nodes of functional memory mice. PCR amplification of Tcf7 locus in IL-7R^lo^ and IL-7R^hi^ genomic DNA (loading control, regular primers). (*B*) Relative quantification of *Tcf7* methylation IL-7R^lo^ and IL-7R^hi^ CD8^+^ T cells (*C*) Relative quantification of genomic DNA/loading control in the IL-7R^lo^ and IL-7R^hi^ conditions. (*D* and *E*) Quantification of *Il7r* (*D*) and *Tcf7* (*E*) expression by RT-PCR in murine T cells upon treatment with vehicle control (black) or hypomethylating agent RG-108 (red). (*F*) Visualization of chromatin accessibility at or near transcriptional start sites (TSS) of indicated genes. Peaks from exhausted phenotype shown in black, functional memory shown in red. See also *SI Appendix*, Fig. S4*A*. (*G*) Diagram of tumor rechallenge experiment in functional memory mice shown in *H* and *I*. (*H*) Bar graphs showing comparison of IFN-g, IL-2, IL-12, and IL-1ß cytokine levels in functional memory (red) and tumor rechallenged conditions (purple). See also *SI Appendix*, Fig. S4 *B* and *C*. (*I*) Quantification of IFN-g, TNF-a and IL-2 production upon stimulation of IL-7R^lo^ (black) and IL-7R^hi^ (red) CD8^+^ T cells from tumor-draining lymph nodes of functional memory mice. (*J*) Kaplan–Meier survival curves (*J*, *Left*) for C57BL/6 mice grafted with melanoma cells after receiving untreated IL-7R^lo^ (dark blue) population, untreated IL-7R^hi^ population (crimson), RG-108-treated IL-7R^lo^ population (light blue), or RG-108-treated IL-7R^hi^ population (red) of CD8 T cells from tdLN of functional memory mice by adoptive transfer (red). Pie charts (*J*, *Right*) showing percent survival in these same groups. (*K*) Scatter plot of TCF7 methylation (beta value) and gene expression (RSEM log2) in cohort of human melanoma samples (72). Each dot represents an individual sample (Pearson’s r = 0.67, *P* = 2.51 × 10^−62^). See also *SI Appendix*, Fig. S4*D*. (*L*) Bar graph of *Tcf7* promoter methylation in cohort of human melanoma samples stratified into an IL-7R^hi^ (red) and IL-7R^low^ (black) group. (*M*) Heatmap showing DNA methylation of IL7R^hi^-defining genes in a large cohort of melanoma patients stratified by IL-7R expression. Hypomethylation shown in blue, hypermethylation shown in red. Student’s *t* test, Log-rank or ANOVA; *P < 0.05, **P < 0.01, ***P < 0.001. Bar graphs shown as mean +/− SEM, based on three biological replicates. Violin plots show the median (dashed line) and quartiles (dotted lines). Cytokines measured by ELISA using recombinant protein standard curve. Cell count based on luminescence of CellTiterGLO v2 assay. Results based on three biological replicates.

To better understand the epigenetic landscape of IL-7R^hi^ CD8^+^ cells, we explored the epigenetic regulation of IL-7R^hi^ CD8^+^-related genes, such as *Il7r* and *Tcf7*. DNA methylation regulates *Il7r* expression in human CD8^+^ T cells (62). Similarly, *Tcf7* methylation can regulate *Tcf7* expression during early effector T cell differentiation (63, 64). We hypothesized that *Tcf7* and *Il7r* expression may also be regulated by DNA methylation in the context of functional memory. We sorted IL-7R^hi^ and IL-7R^lo^ CD8^+^ T cell populations and analyzed Tcf7 promoter methylation by methylation-specific PCR. IL-7R^lo^ cells had significantly higher Tcf7 DNA methylation compared with IL-7R^hi^ cells (Fig. 5 *A* and *B*), suggesting a transcriptionally repressive role. To functionally test the effect of DNA methylation on *Tcf7* and *Il7r* expression, we treated TILs with the hypomethylating agent RG108 ex vivo and measured *Tcf7* expression by quantitative RT-PCR (Fig. 5 *D* and *E*). Treatment with RG108 increased the expression of *Tcf7* twofold (Fig. 5*E*), and similar findings were seen with *Il7r* (Fig. 5*D*).

Promoter hypermethylation is generally associated with stable gene repression (65, 66), while promoter hypomethylation, as seen in IL-7R^hi^ CD8^+^ cells, is typically considered permissive of transcription (67) and can be associated with accessible chromatin (68). To investigate the chromatin accessibility of the Il7r and Tcf7 promoter in functional memory, we quantified open chromatin accessible regions using ATAC-seq (Fig. 5*F* and *SI Appendix*, Fig. S4*A*). *Il7r*, *Tcf7,* and other memory-associated loci showed an open chromatin configuration near their transcriptional start site (TSS) (Fig. 5 *F*, *Upper* *Left*), while genes associated with exhaustion, such as *Pdcd1* and *Havcr2* (Fig. 5 *F*, *Upper* *Right*), had a closed chromatin configuration, in agreement with our single-cell RNA-seq expression data. Interestingly, canonical effector loci associated with cytotoxicity and production of IFN-g, IL-2 and TNF-a (Fig. 5*F* bottom row) as well as *Gzma*, *Gzmb* and *Prf1* retained an open chromatin accessibility region, but were not appreciably expressed. An open chromatin configuration allows binding of transcription factors and gene expression (69). The open chromatin configuration at these loci suggested to us that genes encoding effector functions are poised for transcription and that IL-7R^hi^ cells are functionally primed to respond to tumor rechallenge. Memory-permissive poised epigenetic states have been previously reported in cytokine regulatory elements of antigen-specific (70) and memory populations (71).

To investigate if this open chromatin state has functional significance, we rechallenged functional memory mice with YUMM-OVA or YUMM-GFP33/66 melanoma cells (day 43) and sorted CD8^+^ T cells one day after tumor rechallenge to measure cytokine production (Fig. 5*G* and *SI Appendix*, Fig. S4*B*). Following rechallenge, there was a significant production of effector cytokines, including IFN-g, IL-2, IL-12, and IL-1ß (Fig. 5*H* and *SI Appendix*, Fig. S4*C*). To delineate the contributions of the IL-7R^hi^ population to this poised effector state, we sorted IL-7R^hi^ and IL-7R^lo^ CD8^+^ T cells from tumor-draining lymph nodes of functional memory mice and measured their ability to produce effector cytokines. Strikingly, after stimulation, the IL-7R^hi^ population showed a significantly higher increase in IFN-g, TNF-a, and IL-2 production compared with the IL-7R^lo^ population (Fig. 5*I*).

Given the ability of RG108 to epigenetically induce memory-related transcripts (Fig. 5 *D* and *E*) and based on the above functional studies (Fig. 5*I*), we hypothesized that the epigenome plays critical roles in the ability of IL-7R^hi^ CD8+ to produce antitumor responses (Fig. 4*E*), and that pharmacologic epigenetic agents could further improve their antitumor activity.

To test this possibility, we used the YUMMER1.7 cell line (31), which was generated by exposing the parental YUMM 1.7 cell line to UV radiation to mimic the neoantigen load of human tumors but without a dominant antigen. We generated YUMMER1.7 functional memory mice and adoptively transferred lymph node IL-7R^hi^ or IL-7R^lo^ CD8+ T cells treated ex vivo with hypomethylating agent RG-108 to naive mice. Strikingly, transfer of RG-108-treated IL-7R^hi^ CD8^+^ T cells conferred protective immunity against YUMMER1.7 to naive mice (Fig. 5*J*) (*P* = 0.001, Log-rank test), extending the median survival from 30 d to undefined (>80 d) and led to tumor clearance in 75% of the mice (Fig. 5*J*). Equally important, RG-108 treatment led to increased IL-7R expression (Fig. 5*D*) and abrogated the difference in antitumor efficacy seen between IL-7R^lo^ and IL-7R^hi^ we previously found (Fig. 4*D*), leading to tumor clearance in 60% of the RG-108-treated IL-7R^lo^ T cell recipients (Fig. 5*J*). These findings suggest that the epigenetic state is a key determinant of antitumor function in these cell populations.

To determine the relevance of these findings in human melanoma, we analyzed the pattern of IL-7R expression in the TME of a large cohort of melanoma patients (72). High *Tcf7* promoter methylation in human melanoma samples is significantly associated with low *Tcf7* expression level, suggesting a suppressive role for DNA methylation (Fig. 5*K*). We next stratified the melanoma cohort based on IL-7R expression into an IL-7R^hi^ and IL-7R^lo^ group. Consistent with our experimental findings, the IL-7R^hi^ group exhibited hypomethylation of the Tcf7 promoter and significantly higher Tcf7 expression compared to the IL-7R^lo^ group (Fig. 5*L*). Importantly, we found that the IL-7R^hi^ CD8+ memory signature we identified experimentally in our mouse model, was hypomethylated in the IL-7R^hi^ melanoma group (Fig. 5*M* and *SI Appendix*, Fig. S4*D*), suggesting that DNA methylation likely regulates this memory signature.

Our findings suggest that IL-7R^hi^ CD8+ cells have a distinct, functionally poised epigenetic landscape regulated by DNA methylation, which confers superior antitumor cytotoxicity and can be potentiated by hypomethylating agents to improve cell-based therapies.

### IL-7R Is a Marker of a Memory-Like Population in the TME and a Prognostic Factor for Melanoma Survival.

Based on our observations, we hypothesized that high *IL-7R* expression may be associated with improved melanoma survival and durable response to therapy. We first investigated whether the *IL-7R*^hi^ memory transcriptional signature we identified experimentally in our functional memory model is present in the human melanoma TME.

**Fig. 6.**
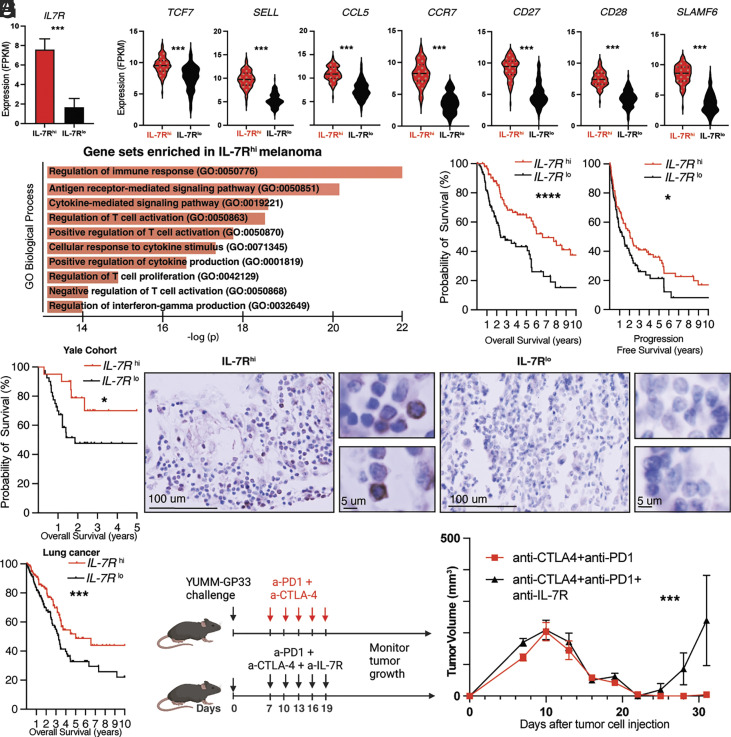
IL-7R is a marker of a memory-like population in the TME and a prognostic factor for melanoma survival. (*A*) Melanoma patient cohort [72] was stratified based on *IL-7R* expression into an *IL-7R*^hi^ (red) and *IL-7R*^lo^ group (black). (*B*) Violin plots comparing expression of indicated genes in the *IL-7R*^hi^ (red) and *IL-7R*^lo^ groups (black) in the melanoma patient cohort. See also *SI Appendix*, Fig. S5*A*. (*C*) Top ten gene sets enriched in the *IL-7R*^hi^ melanoma cohort. See also *SI Appendix*, Fig. S5*B* for detailed statistics. (*D*, *Left*) Kaplan–Meier curve comparing overall survival of the *IL-7R*^hi^ and *IL-7R*^lo^ melanoma groups (n = 96 patients per group). (*D*, *Right*) Kaplan–Meier curve comparing progression-free survival of the *IL-7R*^hi^ and *IL-7R*^lo^ melanoma groups in the same cohort (n = 96 patients per group). (*E*) Kaplan–Meier curve comparing overall survival of the *IL-7R*^hi^ and *IL-7R*^lo^ groups in a Yale cohort of 60 patients with melanoma who received immune checkpoint inhibitors. (*F*) Immunohistochemical staining for IL-7R of pretreatment whole-tissue sections in the Yale melanoma cohort. (*F*, *Left*) Representative image of IL-7R^hi^ staining and accompanying 40× magnification insets. (*F*, *Right*) Representative image of IL-7R^lo^ staining and accompanying 40× magnification insets. (*G*) Kaplan–Meier curve comparing overall survival of *IL-7R*^hi^ and *IL-7R*^lo^ lung cancer groups (n = 140 patients per group). (*H*) Experimental approach to IL-7R blockade. Wild-type C57BL/6 mice received immunotherapy alone (anti-CTLA4 and anti-PD1, shown in red) or immunotherapy with concurrent IL-7R blockade (shown in black) 7 d after melanoma cell injection. (*I*) Tumor volume curves for mice receiving immunotherapy alone (black) or immunotherapy with concurrent IL-7R blockade (red) from indicated groups described in *H*. Statistical tests used: Student’s *t* test or ANOVA and Log-rank test; *P < 0.05, **P < 0.01, ***P < 0.001. Bar graphs shown as mean +/− SEM, based on three experiments (except human data). Violin plots show the median (dashed line) and quartiles (dotted lines). n = 5 mice per group.

We stratified a large cohort of patients with advanced melanoma (72) into an IL-7R^hi^ and IL-7R^lo^ group (Fig. 6*A*) and compared expression of the signature we found experimentally between the *IL-7R*^hi^ and *IL-7R*^lo^ groups. We found that the human *IL-7R*^hi^ melanoma TME group recapitulated the transcriptional signature we experimentally detected in functional memory mice (Fig. 6*B* and *SI Appendix*, Fig. S5*A*). Gene set enrichment analysis of the *IL-7R*^hi^ group identified T cell signaling and immune response pathways, including immune response regulation (*P* = 5.78 × 10^−23^), antigen receptor–mediated signaling (*P* = 2.34 × 10^−21^), cytokine-mediated signaling (*P* = 1.48 × 10^−19^), regulation of T cell activation (*P* = 1.79 × 10^−19^), and regulation of interferon-g production (*P* = 7.56 × 10^−15^) among the top enriched pathways (Fig. 6*C* and *SI Appendix*, Fig. S5*B*). Overall, the pathways enriched in the *IL-7R*^hi^ melanoma TME cohort showed significant overlap with the experimentally identified *IL-7R*^hi^ cells, suggesting that a memory-like transcriptional profile is present in the melanoma TME.

Importantly, the *IL-7R*^hi^ TME group (Fig. 6 *D*, *Left*) had longer overall survival (2,421 d vs. 875 d, *P* < 0.0001, Log-rank test) and progression-free survival (755 vs. 523 d, *P* = 0.03, Log-rank test) (Fig. 6 *D*, *Right*) compared with the IL-7R^lo^ TME group. Expression of *IL7R* did not vary significantly with ulceration, clinical substage or Breslow thickness (*SI Appendix*, Fig. S5*G*). Interestingly, *TCF7* expression was not associated with differences in survival, suggesting that the effect of *IL-7R* is not simply a reflection of CD8^+^ infiltration or a stemness program orchestrated by *TCF7* (*SI Appendix*, Fig. S5 *C* and *D*). We next performed Cox-proportional hazard analysis of *IL-7R* expression in relation to overall survival in the melanoma cohort. Common prognostic clinicopathologic variables, including age, gender, Breslow thickness, clinical stage, and ulceration were used as covariates. In univariate and multivariate analyses, IL-7R expression was an independent prognostic factor of overall survival (HR = 0.91, 95%CR 0.85 to 0.97, *P* = 0.01) (*SI Appendix*, Fig. S5*E*).

To investigate these findings further, we evaluated IL-7R expression by immunohistochemistry in a cohort of 60 patients with melanoma treated with checkpoint inhibitors at Yale. In this independent cohort, high IL-7R expression was associated with significantly longer tumor-specific survival (undefined vs. 678 d, *P* = 0.02, Log-rank test) compared with the IL-7R^lo^ group (Fig. 6 *E* and *F*). TCF7^hi^ was not associated with significantly different survival compared with TCF7^lo^ samples (*SI Appendix*, Fig. S5*F*), in agreement with our prior observations. Beyond melanoma, *IL-7R*^hi^ is also associated with significantly longer overall survival in a large cohort of patients with lung cancer (Fig. 6*G*).

To better understand the association of IL-7R with survival, we next investigated how IL-7R signaling impacts the acute immune response to anti-CTLA4 and anti-PD1 therapy. We grafted wild-type mice with YUMM-GFP33/66 cells, and 7 d later the mice were divided into a group receiving immunotherapy alone (combined anti-CTLA4 and anti-PD1) or immunotherapy with IL-7R blockade (Fig. 6*H*). Blocking IL-7R concurrently with immunotherapy did not affect the remission induced by anti-PD1 and anti-CTLA4 therapy (Fig. 6*I*). Strikingly however, IL-7R blockade enhanced recurrence (Fig. 6*I*), suggesting a critical role for IL-7R signaling in maintaining a durable antitumor immune response.

Collectively, our work identifies IL-7R as a functional marker of a memory/cytotoxic CD8^+^ T cell population, which is critical for functional antitumor immunity after checkpoint therapy or surgery. This tumor-specific population has a distinct epigenome, superior antitumor function, and can be enriched for adoptive cell therapy based on high IL-7R expression, without knowledge of tumor antigens, and may thus help improve melanoma therapy in the postsurgical or postimmunotherapy setting.

## Discussion

Despite significantly improved outcomes with anti-CTLA-4 and anti-PD-1/PD-L1 therapies, approximately half of patients with advanced melanoma do not achieve a durable response and face a high risk of recurrence, for which treatment options are limited. Adoptive transfer is a promising treatment modality for patients with advanced melanoma recurrence (1), but the identity of an optimal T cell population functionally poised to deliver effective antitumor immunity has been poorly defined (73).

Our work, using a model for the induction of antimelanoma memory with a dominant tumor antigen, identifies a population of tumor-specific IL-7R^hi^ memory CD8^+^ T cells that resides in lymphoid organs, plays critical roles in antitumor memory, and has superior antitumor functional capacity. These tumor-specific IL-7R^hi^ cells have a distinct transcriptional program, including T_CM_-like memory markers, cytotoxic properties reminiscent of T_EM_ cells, and notably lack exhaustion markers. The formation of these long-lived cells is necessary to maintain antitumor immunity in the context of checkpoint blockade or surgical excision. The IL-7R^hi^ memory/cytotoxic population is found in the tumor-draining lymph node and spleen early after priming and is functionally dependent on IL-7R signaling. Blocking the IL-7 receptor abolishes the establishment of the population and results in prevention of antitumor memory. Our studies use clinically relevant immunogenic melanoma lines that have the capacity to respond to checkpoint inhibitor therapies. Whether the T cell population we describe also plays important roles in less immunogenic melanomas is subject to future research.

Enriching for tumor-specific T cells for adoptive transfer in the clinical setting remains challenging (74). TILs commonly used for adoptive transfer include only a small fraction of tumor-specific cells, also include suppressive T cell populations (10), largely lack memory/stemness properties (12, 13) and commonly express markers of exhaustion, (17, 18), limiting their functional antitumor activity. The tumor-draining lymph node is a potential source of T cells with a memory/stemness program that lack exhaustion and has been proposed in other cancer types (75). Importantly, we show that >95% of tumor-specific CD8^+^ T cells in the postimmunotherapy tumor-draining lymph node express high levels of IL-7R, which is otherwise sparsely expressed in the lymph node by nonantigen-specific cells. Selection for IL-7R^hi^ could be a strategy to indirectly enrich for a tumor-specific CD8+ population without known tumor antigens. Tumor-draining lymph node tissue is available for a subset of advanced melanoma patients (usually intermediate thickness melanomas T2/T3 corresponding to Stage III substages) as part of melanoma clinical management (76). Functionally, IL-7R^hi^ CD8^+^ T cells have superior antitumor activity compared to their IL-7R^lo^ counterparts. Transfer of the IL-7R^hi^ CD8^+^ population significantly decreases tumor growth, prolongs survival, and leads to tumor clearance in a subset of naive mice. In addition to constitutive intrinsic cytotoxic properties, these cells have a functionally poised chromatin landscape without epigenetic “scars” that allows them to rapidly recall effector function, such as production of IFN-g and IL-2. DNA methylation plays a prominent role in their epigenetic identity and hypomethylating agents can induce and IL-7R^hi^ state ex vivo and significantly potentiate antitumor function. Alterations in DNA methylation, through TET2 and DNMT3A, have been implicated in regulating CAR-T cell stemness/exhaustion and antitumor activity (77, 78), but are incompletely understood.

Integrating and contextualizing our findings with studies from human melanoma, we found that an IL-7R^hi^ memory signature is present in the melanoma TME. Our findings agree with data reported by Sade-Feldman et al. who identified a CD8^+^ cluster with increased expression of genes linked to memory and associated with improved immune response (79). Interestingly, our data suggest that blocking IL-7R does not preclude response to checkpoint inhibition acutely (Fig. 6*I*), but IL-7R signaling is required to maintain a response to therapy. A recent study reported that anti-PD-1 response and melanoma patient survival is associated with a late T cell memory transcriptional profile (80). They identified a responder-associated single-cell cluster with increased IL-7R expression, corresponding to long-lived memory T cell programming, in agreement with our findings. We revealed that IL-7R is an independent prognostic factor of survival in melanoma and other malignancies.

These findings advance our basic understanding of antitumor memory in the context of checkpoint inhibition or surgical resection, and suggest a strategy of using high IL-7R expression to enrich for memory T cells with superior antitumor activity from the tumor-draining lymph node, which can be augmented by epigenetic therapies for adoptive cell transfer. Adoptive cell transfer classically relies on TILs, which are largely terminally differentiated, exhausted, and include immunosuppressive populations. The lymph node IL-7R^hi^ cells with cytotoxic/memory properties and a permissive “unscarred” epigenome are prime candidates for adoptive T cell transfer therapies and can help improve T cell–based immunotherapy.

## Materials and Methods

### Animal Experiments.

Mice were maintained at Yale University in accordance with Institutional Animal Care and Use Committee guidelines. Mouse strains were purchased from Jackson Labs, including WT C57BL/6 (#000664), P14 TCR transgenic mice (#037394), RAG KO mice (#002216). 1 to 20 × 10^5^. Tumors were injected subcutaneously into the flanks of 8-wk-old, age-matched C57BL/6J mice in 100 μL of PBS (GIBCO). Tumor size was measured using an electronic caliper. FTY720 (#S5002, Selleck Chemicals) was diluted in PBS and mice were injected with 3 mg/kg three times weekly for the duration of the experiment. For CD8+ depletion experiments, mice were treated twice weekly with 200 μg of anti-CD8 antibody (*SI Appendix*).

### Cell Lines and Plasmids.

Cells were grown at 37 °C and 5% CO2, in DMEM F-12 or Opti-Mem (GIBCO) media supplemented with heat-inactivated Fetal Bovine Serum (Sigma) and Pen/Strep (GIBCO). YUMM-OVA was a kind gift from Dr. Ping-Chih Ho (University of Lausanne). YUMM-GFP33/66 was generated by Gibson cloning into a lentiviral backbone, transfecting 293FS* cells harvesting virus titer and transducing YUMM1.7 an MOI of two followed by selection for GFP expression by FACS (Sony SH800). MC38 was purchased from Kerafast Biotech. Sequences available in *SI Appendix*, Fig. S6.

### Adoptive T Cell Transfer.

Murine melanoma tumors, lymph nodes, or spleens were processed into single-cell suspensions, stained with antibodies against mouse CD45, CD3, CD8, gp33-tetramer, CD127, and sorted using a BD Aria Cell Sorter (BD Biosciences). Sorted T cells (10,000 to 100,000) were reconstituted in PBS (Sigma) and a final volume of 100 μL was injected retroorbitally. Unless otherwise specified in figure legend, 1 × 10^4^ CD127^+^ and 1 to 6 × 10^4^ CD127^-^ cells were transferred.

### ELISA.

IFN-gamma, IL-2 and TNF-a ELISAs were performed according to the manufacturer’s instructions using ELISA kits (R&D systems, DY402-05, DY410-05; Biolegend 430801) and the Mouse Cytokine Array Discovery Assay (EVE Technologies).

### Bisulfite Modification, qPCR and Methylation-Specific PCR.

Of note, 1 μg of genomic DNA per sample was bisulfite converted using the Zymo EZ DNA Methylation Kit (Zymo Research). Methylation-specific PCR primers were designed using BiSearch and conversion performed on a C1000 Touch Thermal Cycler (BioRad). with ZymoTaq PreMix and HotStart Polymerase on a C1000 Touch Thermal Cycler (BioRad). For qRT-PCR, total RNA was extracted using TRIzol (Qiagen) and the RNeasy Plus mini kit (Qiagen). cDNA was made using SuperScript IV Reverse Transcriptase (Thermo Fisher Scientific) on a CFX96 Real-Time PCR System (Bio-Rad) and iTaq Universal Probes Supermix (Bio-Rad) and sequence-specific oligonucleotide primers purchased from Sigma-Aldrich. Expression values were calculated using the standard curve method.

### Flow Cytometry Analysis.

Fluorescence spectra were acquired using a BD LSRII or BD Symphony (BD Biosciences) flow cytometers and analyzed by FlowJo (Version 10, BD). For flow cytometry analysis, splenocytes and/or fluorescent minus one staining was used for gating. Sorting was performed at the Yale Flow Cytometry Core on a BD FACSAria instrument.

### CD8^+^ T Cell Enrichment.

CD8^+^ T cells were purified from single-cell suspension using the CD8a+ T Cell Isolation Kit (Miltenyi Biotech) according to the manufacturer’s instructions. Murine melanoma lymph were processed into single-cell suspensions as described above, and 10^3^ to 10^8^ cells were incubated with an antibody cocktail (#130-095-236) and conjugated to magnetic beads (Miltenyi Biotech). The CD8+ fraction was allowed to elute by gravity, and the column was washed with 6 mL of wash buffer.

### Single-Cell RNA-Seq.

Tumors or lymph nodes were dissociated and processed into single-cell suspensions and sort-purified: P1: Tetramer^+^CD45^+^CD3^+^ (antigen-specific T cells), P2: CD45^+^CD3^+^Tetramer^−^ (polyclonal T cells), P3: CD45^+^CD3^−^ (Non-T cell immune cells), and P4: CD45^−^. P1, P2, P3, and P4 were mixed at a 2:1:1:1 ratio and cells were encapsulated into droplets using 10x Chromium GEM and libraries prepared using the Single Cell 5′ Reagent Kit version 2.0 (10× Genomics) prior to sequencing using a NovaSeq instrument, as previously described (81).

### ATAC-Seq.

Mouse lymph node tissue was processed into single-cell suspensions, and 50,000 cells were tagmented and processed following the manufacturer’s protocol (ActiveMotif). Indexed libraries were prepared and sequenced to at least 30 × 10^7^ reads on a NovaSeq instrument, as previously described (82).

### Bulk RNA-Seq.

RNA was isolated using the Qiagen RNeasy Plus mini kit and QC was performed on an Agilent 2,200 TapeStation. RNA with satisfactory RIN values were used for stranded library preparation and sequenced with the target of at least 3 × 10^7^ reads per sample. Reads were quality filtered and trimmed of Illumina adapters using FastQC and Cutadapt. Filtered reads were aligned to referen genome mm10 using STAR aligner and quantified using featureCounts. Differential expression analysis was performed with DESeq2.

### Immunohistochemistry.

Tissue microarrays were a gift from H.K. The Yale melanoma cohort sample collection was approved by the Yale Human Investigation Committee protocol in accordance with the Declaration of Helsinki. Antigen retrieval was performed in Target Retrieval Solution (Dako), and slides were incubated with primary antibodies against IL-7R (LS-B2830-50 LSBio), Tcf7 (2203S Rabbit mAb), or CD3 (Cell Signaling Technology 99940S). After washing, slides were incubated with biotinylated secondary antibody and Vectastain ABC kit (Vector Labs), developed using a di-amino-benzidine-peroxidase substrate kit (Vector Labs). Scoring was performed by a board-certified pathologist (HM).

### Statistics and Reproducibility.

Statistical analyses were conducted using R v4.0.2 and Prism 7 (GraphPad). IHC staining and qPCR analyses have been repeated at least twice. At least five mice were used per experimental group. Log-rank (Mantel–Cox) tests were used for tumor survival curve statistical analyses. *P*-values for all qPCR were calculated with two-sided Student’s *t* test. Benjamini–Hochberg or Bonferroni correction was used for multiple statistical comparisons.

## Supplementary Material

Appendix 01 (PDF)

## Data Availability

All data supporting the findings of this study are available within the Article, the *SI Appendix* Data and the NCBI Sequence Read Archive (SRA) repository (PRJNA902911) (83). All study data are included in the article and/or *SI Appendix*. Previously published data were used for this work (72).
