# Supplementary material for: IL-7R licenses a population of epigenetically poised memory CD8+ T cells with superior antitumor efficacy that are critical for melanoma memory
Source: Proc Natl Acad Sci U S A. 2023 Jul 17;120(30):e2304319120. doi: 10.1073/pnas.2304319120 (PMC10372654; doi:10.1073/pnas.2304319120)
Supplement: Supplementary file 1 — Appendix 01 (PDF) [file pnas.2304319120.sapp.pdf]

PNAS

Supporting Information for
IL-7R licenses a population of epigenetically poised memory CD8$^+$ T-cells with superior antitumor efficacy that are critical for melanoma memory

Goran Micevic[1,2,*], Andrew Daniels[1,3,*], Karine Flem-Karlsen[2], Koonam Park[2], Ronan Talty[3], Meaghan McGeary[3], Haris Mirza[1,3], Holly N. Blackburn[1,7], Esen Sefik[1],Julie F. Cheung[1], Noah I. Hornick[1], Lilach Aizenbud[4,8], Nikhil S. Joshi[1], Harriet Kluger [4,6,8], Akiko Iwasaki[1,6,9], Marcus W. Bosenberg[1,2,3,4,5,6, #], Richard A. Flavell[1,4,9, #]

[#]Corresponding authors:

Richard Flavell, Department of Immunobiology, Yale School of Medicine, 300 Cedar Street, Ste Suite 569A, New Haven, CT 06519
Phone: 203-785-7024; E-mail: richard.flavell@yale.edu

Marcus W. Bosenberg, Department of Dermatology, Yale School of Medicine, 15 York St, New Haven 06520
Phone: 203-737-4384; E-mail: marcus.bosenberg@yale.edu

This PDF file includes:

Supplemental Methods
Legends for Figures S1 to S6
SI References

Supplemental Methods

Antibodies

The following antibodies were used:

CD3E(152309, 100204, Biolegend; 612803, 564009 BD), CD45 (107632, 103133, 748371,103106, 103155, 147709, 103147, 103124, 564279 Biolegend), CD8A (100759, 100748 Biolegend), CD4 (100526,116016,563790,100406 Biolegend) CD45.1 (565212 BD), CD45.2 (612779, 565390 BD), CD127 (135014, 135052 Biolegend, 562419 BD) TCF1 (655204 Biolegend, 6444 CST), PD-1 (135221, 135218, Biolegend), CD16/32 (101302 Biolegend), Tim-3 (119721, Biolegend), Ifng (505801, Biolegend), CD172a(144006 Biolegend), Ly108 (134610 Biolegend, 561547 BD), CD90.2 (741702 BD). Antibody tetramers to OVA peptide and gp33-41 peptide were generated by the NIH Tetramer Core Facility (TCF) at Emory University. Live/dead fixable aqua dead cell stain kit (L34965).

Bisulfite modification, qPCR and methylation specific PCR

For bisulfite modification, 1µg of genomic DNA extracted from tumors or lymph nodes was performed using the Zymo EZ DNA Methylation Kit (Zymo Research) according to manufacturer's recommended procedures. For methylation specific PCR, primers were designed using BiSearch (http://bisearch.enzim.hu). Conversion was performed using a C1000 Touch Thermal Cycler (BioRad). Amplification was performed with ZymoTaq PreMix and HotStart Polymerase on a C1000 Touch Thermal Cycler (BioRad) (95℃ 10min, 40 cycles of 95℃ 30 sec, 50-65℃ 40 sec, 72℃ for 40 sec, 72℃ for 7 minutes and 4℃ hold) and gel image captured on a ChemiDoc Imaging system (BioRad) for EtBr stained gels. Positive control mouse DNA methylated standard was created by treating genomic DNA isolated from C57BL/6 mice with M.SssI methyltransferase. Negative and positive control methylation standards were purchased from Sigma Inc. and Zymo Research. For qRT-PCR, total RNA was extracted from tissues using TRIzol (Qiagen) and the RNeasy Plus mini kit (Qiagen). cDNA synthesis was performed using SuperScript IV Reverse Transcriptase (Thermo Fisher Scientific). qRT-PCR was performed using a CFX96 Real-Time PCR System (Bio-Rad) and iTaq Universal Probes Supermix (Bio-Rad). Sequence-specific oligonucleotide primers were purchased from Sigma-Aldrich. The following primers were used: *Il7r* (5'-CACCATTCTGAGTTTGTTCTC-3' and 5'-GAGTTTTCTTATGATCGGGG-3') and *Tcf7* (5'-AGGCCAAGTACTATGAACTG-3' and 5'-TCTTCTTTCCGTAGTTATCCC-3'). Expression values were calculated using the standard curve method.

Single Cell Analysis

Raw sequencing reads were processed with Cell Ranger using the mm10 mouse reference genome to generate a gene cell count matrix. Seurat was used to load the matrix and perform analyses. Specifically, this matrix was filtered, retaining cells with more than 200 and fewer than 5,000 genes and less than 5% mitochondrial transcripts. We then normalized and log-transformed expression values. Two thousand of the most variable genes were selected using the FindVariableGenes command and principal component analysis was performed. To visualize the cell subpopulations in two dimensions, we applied *t*-distributed stochastic neighbor embedding (*t*-SNE), a non-linear dimension reduction method, to the log-transformed data using 30 principal components. Graph-based clustering was used to generate clusters that were overlaid on the *t*-SNE coordinates to investigate cell subpopulations. CD8+ T-cells were identified based on Cd3e and Cd8a expression. Subsetting was performed using the command SubsetData. Marker genes for each cluster of cells were identified using the Wilcoxon test with Seurat. For adjusted *P* values, the Bonferroni correction was used.

ATAC-Seq analysis

Read quality was checked using FastQC and MultiQC. Reads were trimmed of adaptor sequences (Trimgalore) and aligned to reference genome mm10 using Bowtie2/BWA_MEM. Orphan and duplicate reads were identified by Picard and removed. Reads were merged using SAMtools and consensus peaks were identified, quantified and annotated using MACS2. For differential accessibility analysis, reads in the consensus peak set were counted for each replicate and analyzed with featureCounts and DESeq2. sequence motifs enriched in gained ATAC-seq regions, significant, differential (adjusted p-value < 0.05) ATAC-seq regions that overlap the noted conditions were used as foreground, and all other ATAC-seq regions within the consensus peak

set were used as background. Versions of software used: Nextflow v22.04.3, FastQC v0.11.9, Trim Galore! v0.6.4_dev, BWA v0.7.17-r1188, Samtools v1.10, BEDTools v2.29.2, BamTools v2.5.1, deepTools v3.4.3, Picard v2.23.1, R v3.6.2, Pysam v0.15.3, MACS2 v2.2.7.1, ataqv v1.1.1, featureCounts v2.0.1, Preseq v2.0.3, MultiQC v1.9.

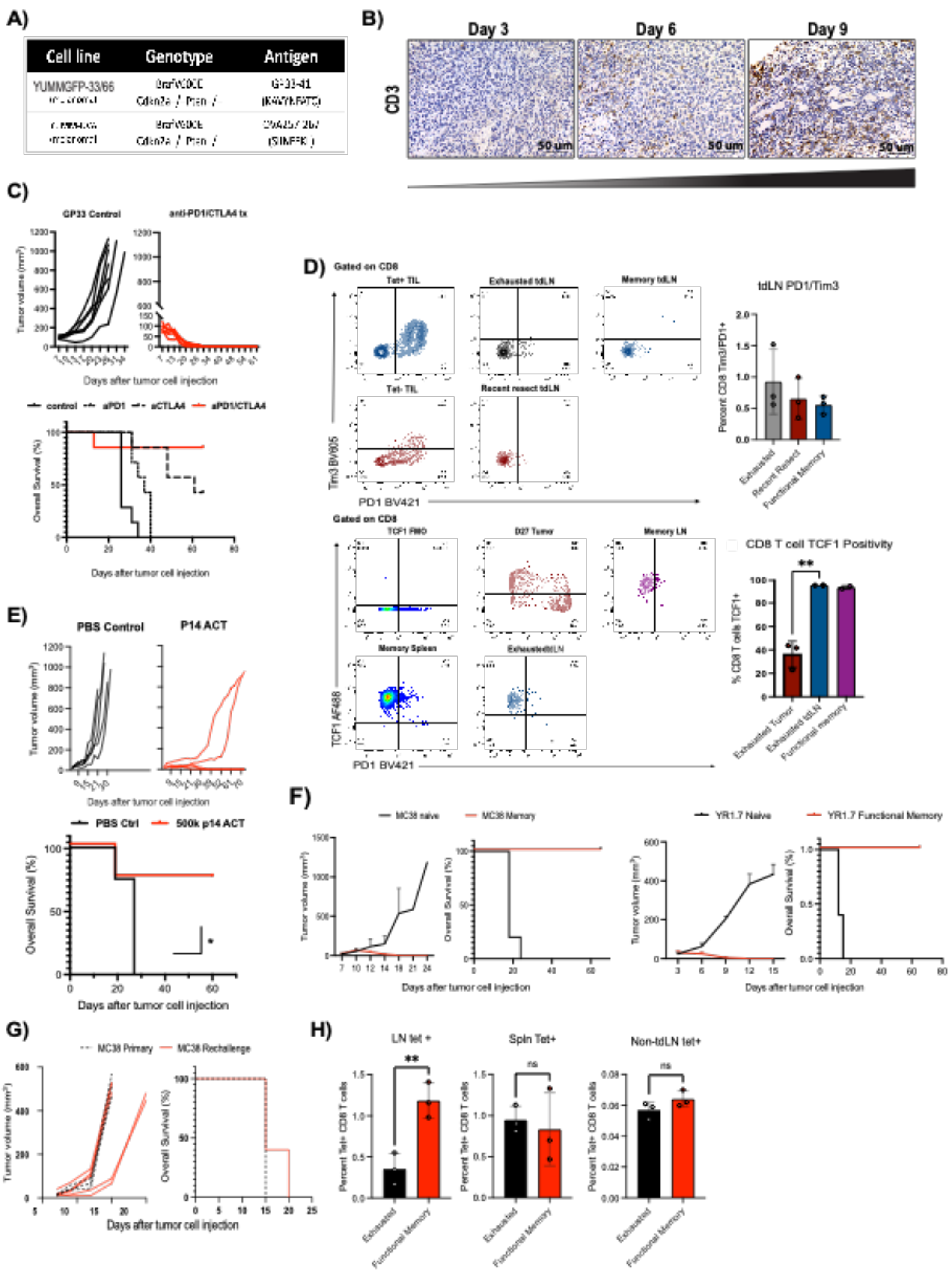

Fig. S1. YUMM-GFP33/66 tumor background, validation of immunogenicity and immune infiltration, and validation of memory induction model in alternate tumor line, related to Figure 1.

A) Driver mutations and dominant antigens in YUMM-GFP33/66 (top) and YUMM-OVA (bottom) tumor lines

B) Immunohistochemistry of mouse YUMM-GFP33/66 tumors at days 3, 6, and 9 using anti-CD3 antibody to assess T cell infiltrate.
C) Growth of tumors in mice engrafted with $1x10^6$ YUMM-GFP33/66 cells and treated either with isotype control IP (left, black) or combination anti-PD1/anti-CTLA4 (right, red). Kaplan-Meier comparing survival of mice engrafted with $1x10^6$ YUMM-GFP33/66 cells and treated either with isotype control IP (solid, black), anti-PD-1 (dotted black), anti-CTLA-4 (dashed black) or combination anti-PD-1/anti-CTLA-4 (solid red).
D) Flow cytometry gating of Tim3/PD1$^+$ CD8$^+$ T-cells (top) in tumor as well as tdLN. Percent Tim3/PD1$^+$ CD8$^+$ T-cells in tdLN of exhausted, 3 days post tumor resection (recently resected), and functional memory mice. Flow cytometry gating of TCF1+ CD8+ T-cells (Bottom) in tumor as well as tdLN. Percent Tim3/PD1+ CD8+ TILs in exhausted mice, in tdLN 10 days post engraftment (exhausted), and tdLN of functional memory mice (p=0.0059, Student's t-test)
E) Tumor growth curves (top) and Kaplan-Meier curves (bottom) of C57BL6 mice receiving ACT with 500k P14 cells (red) or PBS (black) 3 days prior to engraftment with $1x10^6$ YUMM-GFP33/66 tumor cells.
F) Tumor growth and Kaplan-Meier curves of mouse adenocarcinoma cell line, MC38 (left), and melanoma cell line, YUMMER1.7 (right), engraftment in functional memory mice (red) versus age-matched naive controls (black). Functional memory mice were generated using the model in Figure 1A.
G) Tumor growth curves (left) and Kaplan-Meier curves (right) of YUMM-GFP33/66 memory mice (red line) and naïve mice (black line) engrafted with $1x10^6$ mouse adenocarcinoma cell line, MC38 tumor cells.
H) Percent tetramer positive CD8$^+$ T-cells in the tdLN, spleen and contralateral inguinal LN (non-tdLN) of day 14 tumor bearing mice (exhausted) and functional memory mice (p=0.0075, Student's t-test).

Figure S2

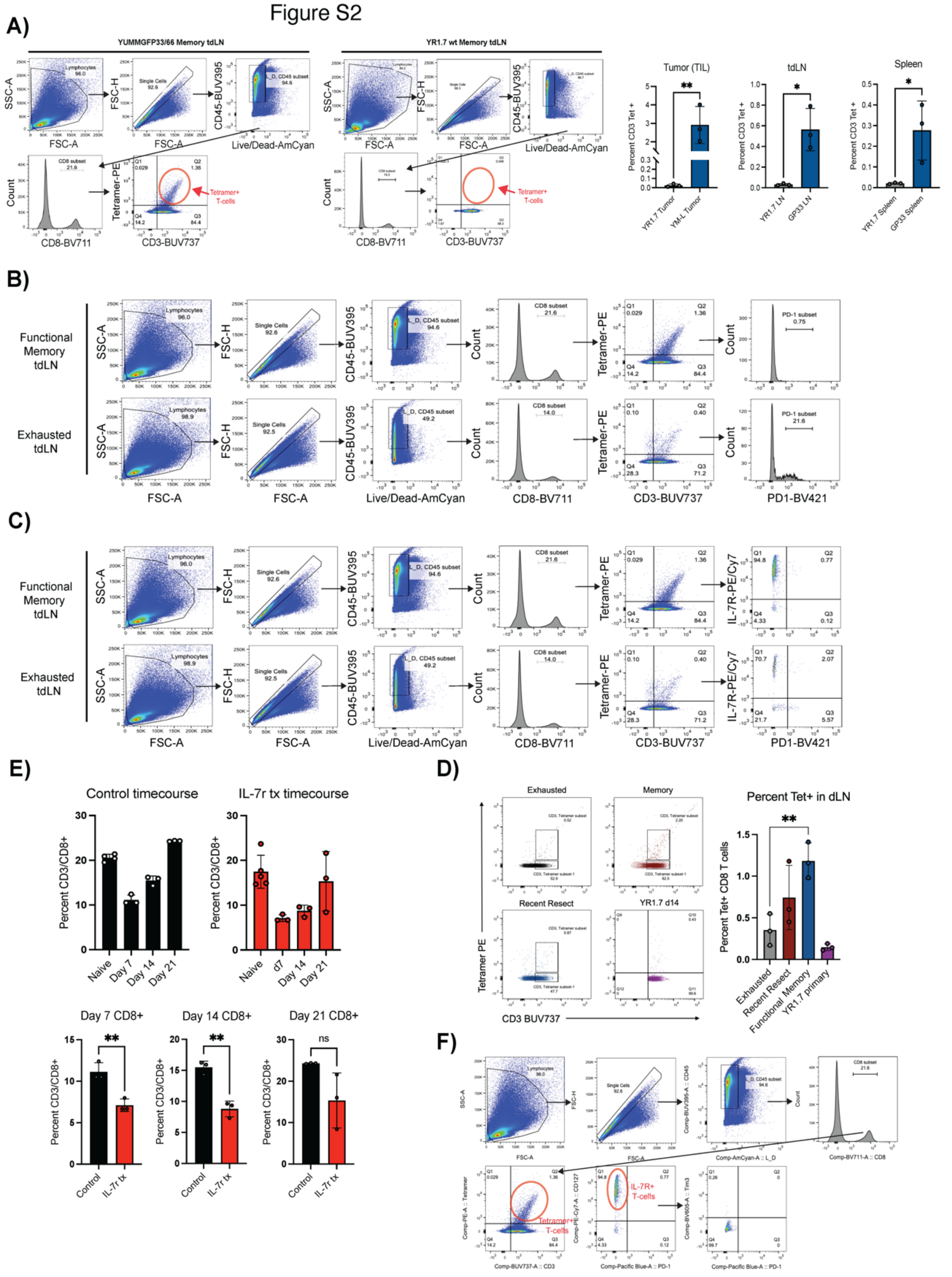

Fig. S2. Flow cytometry gating strategy for exhaustion and stem-like markers, tdLN tetramer positivity and IL-7R blockade time course, related to Figure 2

A) Gating strategy for tetramer positive T cells in tdLN of mice challenged with YUMM1.7-GFP33/66 (dominant antigen containing, far left) and YUMMER1.7 (no dominant antigen, middle)

and bar graphs showing percent tetramer positive T cells TIL (p=0.007), tdLN (P=0.019), and spleen (p=0.03) of mice with YUMMER1.7 (no dominant antigen) (grey) or YUMM-GFP33/66 (blue) (far right).
B) Gating strategy for the assessment of antigen-specific activated/exhausted $CD8^+$ T-cells in tdLN. Functional memory (top) and exhausted (bottom)
C) Gating strategy for the assessment of antigen-specific stem-like $CD8^+$ T-cells in tdLN. Functional memory (top) and exhausted (bottom)
D) Flow cytometry gating (left) and graph of percent tetramer positive $CD8^+$ T-cells (right) in tdLN of day 14 tumor mice (exhausted, grey), day 3 post-resection mice (nascent memory, red), functional memory (blue) and non-GFP33/66 expressing tumor bearing mice (YR1.7, purple). (p=0.007, Student's t-test).
E) Assessment of $CD8^+$ T cell population in tdLN of WT B6 mice prior to and 7, 14, and 21 days post YUMM-GFP33/66 tumor resection (black); as well as IL-7R blocking mAB treated mice at these same time points (red). Trend of the $CD8^+$ T-cell population in each group (top) and comparison across the treatment groups at each day post-resection (bottom). (p=0.0064, 0.0020, 0.0800, respectively. Student's t-test).
F) Gating strategy for the assessment of exhaustion marker expression on IL-$7R^+$ antigen-specific $CD8^+$ T-cells in functional memory tdLN. Representative sample shown lacking Tim3/PD1 expression.
Statistical tests used: Student's t-test or ANOVA and Log-rank test; * p< 0.05, **p<0.01, ***p<0.001.

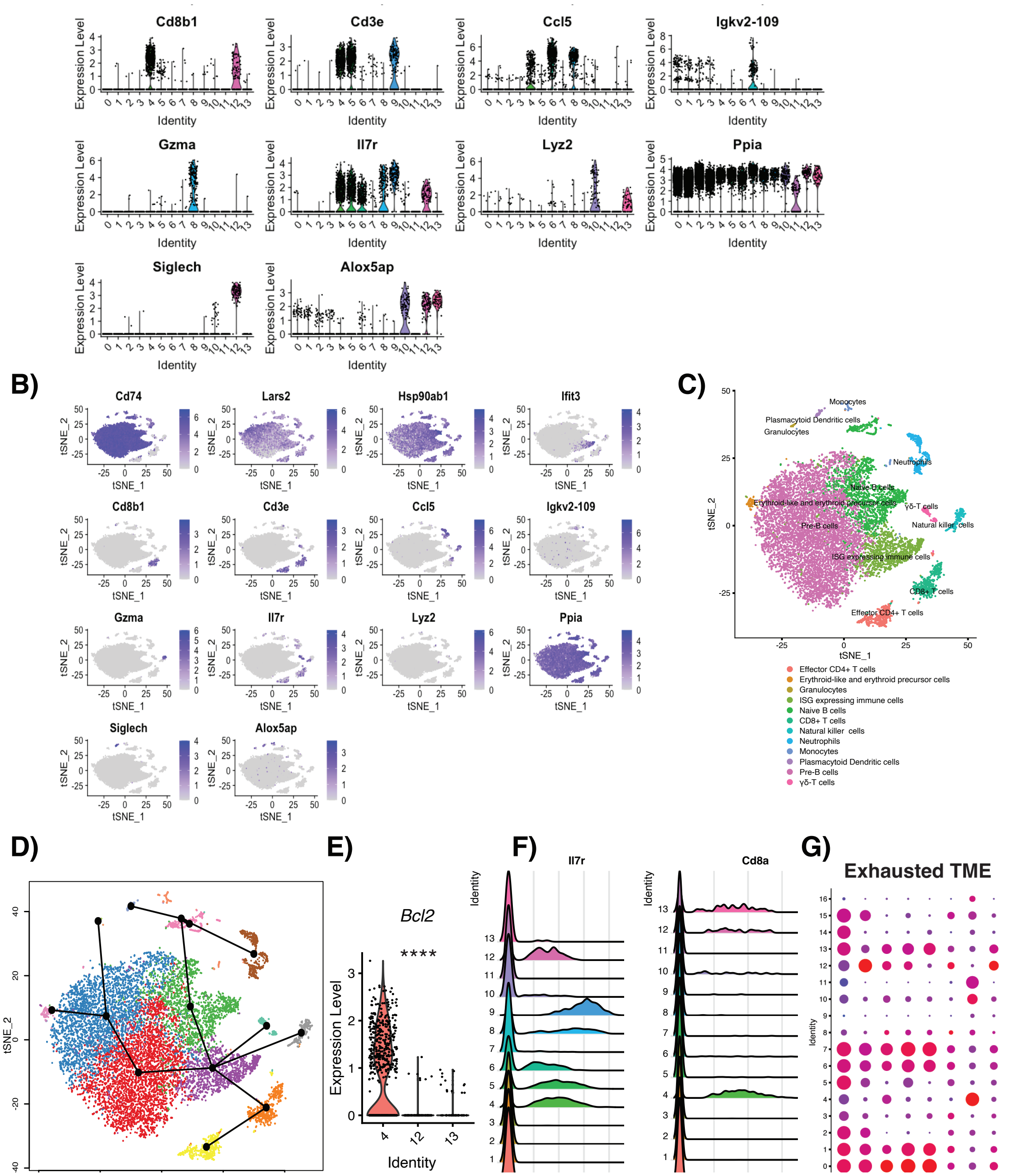

Figure S3. ***IL-7R marks a tumor specific T cell population with central memory-like features*, related to Figure 3.**

A) Violin plot showing expressing and distribution of marker gene expression for each cluster across the dataset. Related to Figure 3C.

B) Single cell transcription level and distribution of expression for indicated genes in the tSNE plot from Figure 3A). Level of transcription displayed as gradient from gray (no expression) to purple (expression).

C) Cluster annotation using automated immune gene canonical markers.

D) Pseudo time analysis of relatedness between different clusters identified.

E) Violin plot showing Bcl2 expression in CD8+ clusters 4, 12 and 13.

F) Ridge plot of CD8 and IL-7R expression across the 13 cell clusters.

G) Dot plot showing relative mean expression level of markers of exhaustion from the melanoma TME of exhausted phenotype 21 days after tumor cell injection (n=5,192 cells).

Statistical tests used: Student’s t-test or ANOVA; * $p < 0.05$, ** $p<0.01$, *** $p<0.001$.

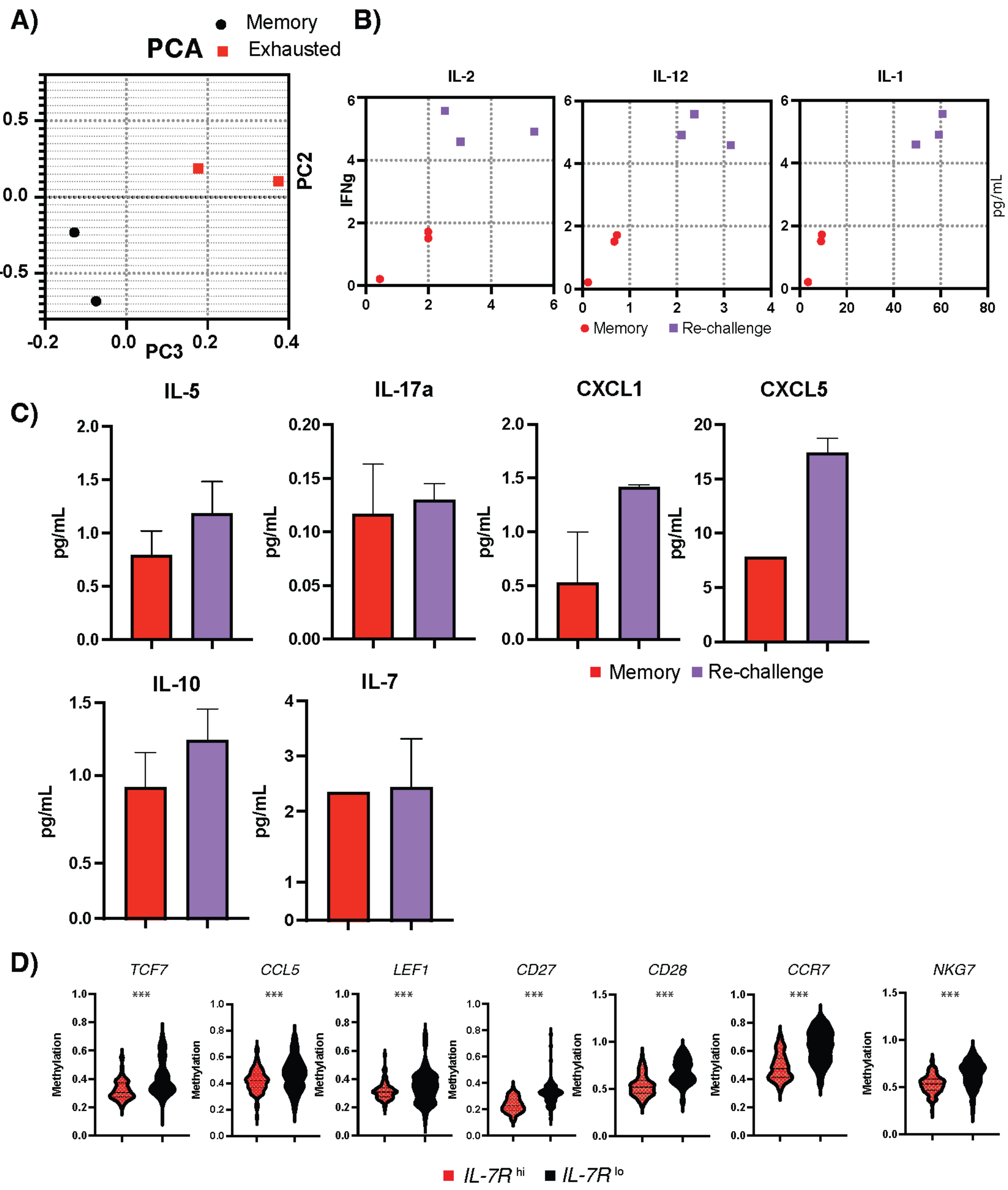

Figure S4. *IL-7R^hi^ CD8+ cells have a poised epigenetic landscape with superior anti-tumor activity*, related to Figure 5.

A) Principal component analysis of functional memory (black) and exhausted phenotype (red) ATAC-Seq samples.

B) Comparison of IFN-g, IL-2, IL-12 and IL-1ß cytokine levels in functional memory (red) and tumor re-challenged conditions (purple). See also Figure 5I.

C) Comparison of indicated cytokine level between functional memory (red) and tumor re-challenge (purple) conditions.

D) Violin plots comparing methylation of indicated genes between the IL-7R^{hi} (red) and IL-7R^{lo} (black) groups in a large melanoma patient cohort.

Statistical tests used: Student's t-test or ANOVA; * $p < 0.05$, ** $p<0.01$, *** $p<0.001$. Benjamini-Hochberg correction for multiple tests.

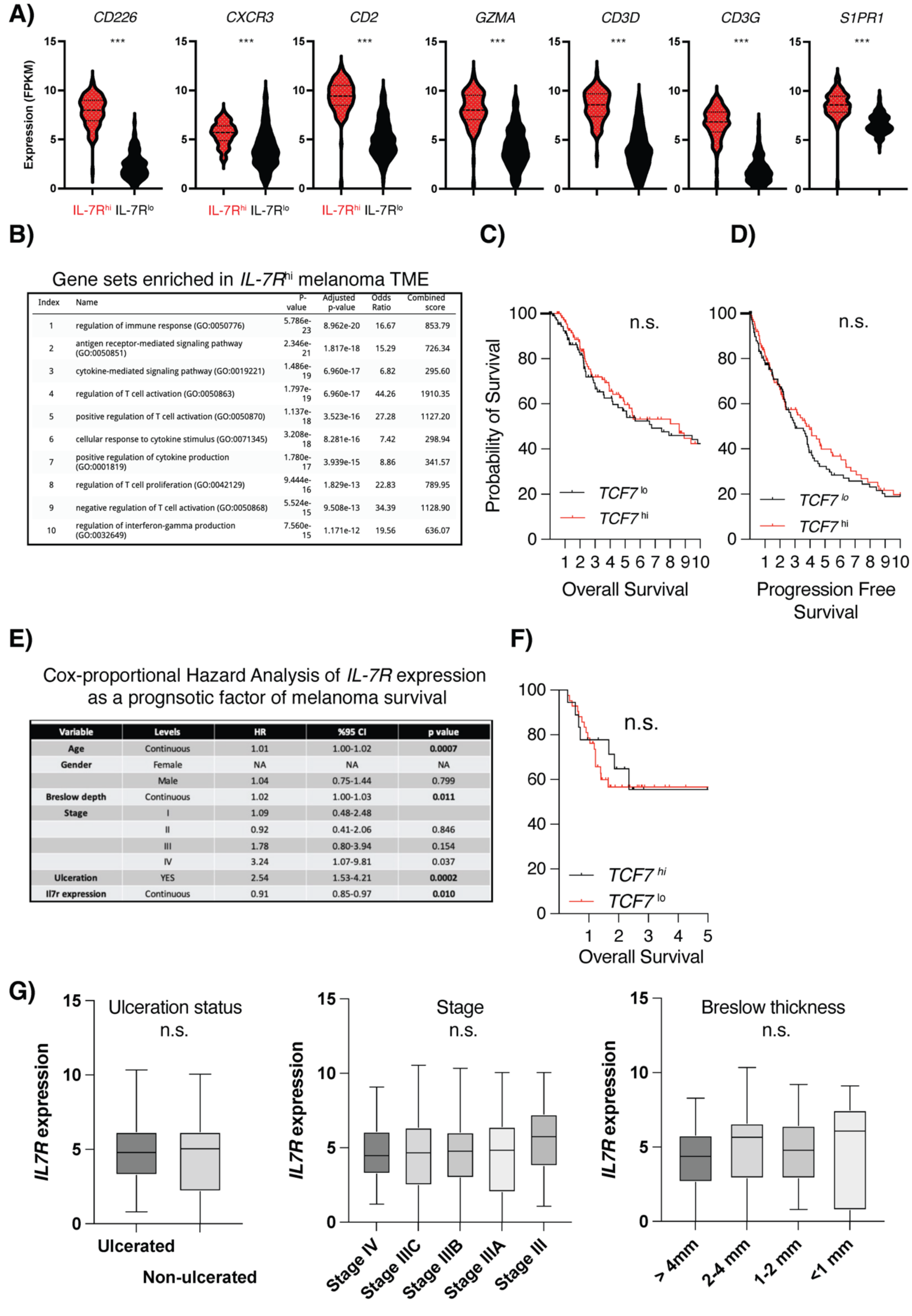

Gene sets enriched in *IL-7R*hi melanoma TME

Index	Name	P-value	Adjusted p-value	Odds Ratio	Combined score
1	regulation of immune response (GO:0050776)	5.786e-23	8.962e-20	16.67	853.79
2	antigen receptor-mediated signaling pathway (GO:0050851)	2.346e-21	1.817e-18	15.29	726.34
3	cytokine-mediated signaling pathway (GO:0019221)	1.486e-19	6.960e-17	6.82	295.60
4	regulation of T cell activation (GO:0050863)	1.797e-19	6.960e-17	44.26	1910.35
5	positive regulation of T cell activation (GO:0050870)	1.137e-18	3.523e-16	27.28	1127.20
6	cellular response to cytokine stimulus (GO:0071345)	3.208e-18	8.281e-16	7.42	298.94
7	positive regulation of cytokine production (GO:0001819)	1.780e-17	3.939e-15	8.86	341.57
8	regulation of T cell proliferation (GO:0042129)	9.444e-16	1.829e-13	22.83	789.95
9	negative regulation of T cell activation (GO:0050868)	5.524e-15	9.508e-13	34.39	1128.90
10	regulation of interferon-gamma production (GO:0032649)	7.560e-15	1.171e-12	19.56	636.07

Cox-proportional Hazard Analysis of *IL-7R* expression as a prognsotic factor of melanoma survival

Variable	Levels	HR	%95 CI	p value
**Age**	Continuous	1.01	1.00-1.02	**0.0007**
**Gender**	Female	NA	NA	NA
	Male	1.04	0.75-1.44	0.799
**Breslow depth**	Continuous	1.02	1.00-1.03	**0.011**
**Stage**	I	1.09	0.48-2.48	
	II	0.92	0.41-2.06	0.846
	III	1.78	0.80-3.94	0.154
	IV	3.24	1.07-9.81	0.037
**Ulceration**	YES	2.54	1.53-4.21	**0.0002**
**Il7r expression**	Continuous	0.91	0.85-0.97	**0.010**

Figure S5. IL-7R in the melanoma TME is associated with improved survival, related to Figure 6.

A) Violin plots comparing expression of indicated IL-7R^{hi} marker genes between the IL-7R^{hi} (red) and IL-7Rlo (black) groups in a large melanoma patient cohort. Related to Figure 6A and B.
B) Top enriched gene sets identified in the functional memory phenotype, related to Figure 6C.
C-D) Kaplan-Meier plots comparing overall survival (C) and progression-free survival (D) of melanoma patients stratified by TCF7 expression (high in red, low in black) in a large cohort of melanoma patients (51). N=96 per group.
E) Multivariable Cox-proportional hazard analysis of IL-7R expression in relation to overall survival in melanoma cohort HR 0.91 (95% CI 0.85-0.97). Age, gender, Breslow depth, clinical stage and ulceration status were used as co-variates. IL-7R expression is an independent predictor of melanoma-specific overall survival with a protective effect HR 0.91 (95% CI 0.85-0.97).
F) Kaplan-Meier curve comparing the overall survival of melanoma patients from a Yale cohort (n=60) stratified into TCF7^{hi} (red) and TCF7^{low} (black) groups. n.s. p>0.05 Log-rank test.
G) *IL-7R* expression did not vary significantly with Breslow thickness, clinical substage or ulceration status in metastatic melanoma cohort (n=199) from TCGA. Data shown as mean and 5-95 percentile range. n.s. p>0.05 ANOVA.
Statistical tests used: Student's t-test or ANOVA and Log-rank test; * p< 0.05, **p<0.01, ***p<0.001.

A)

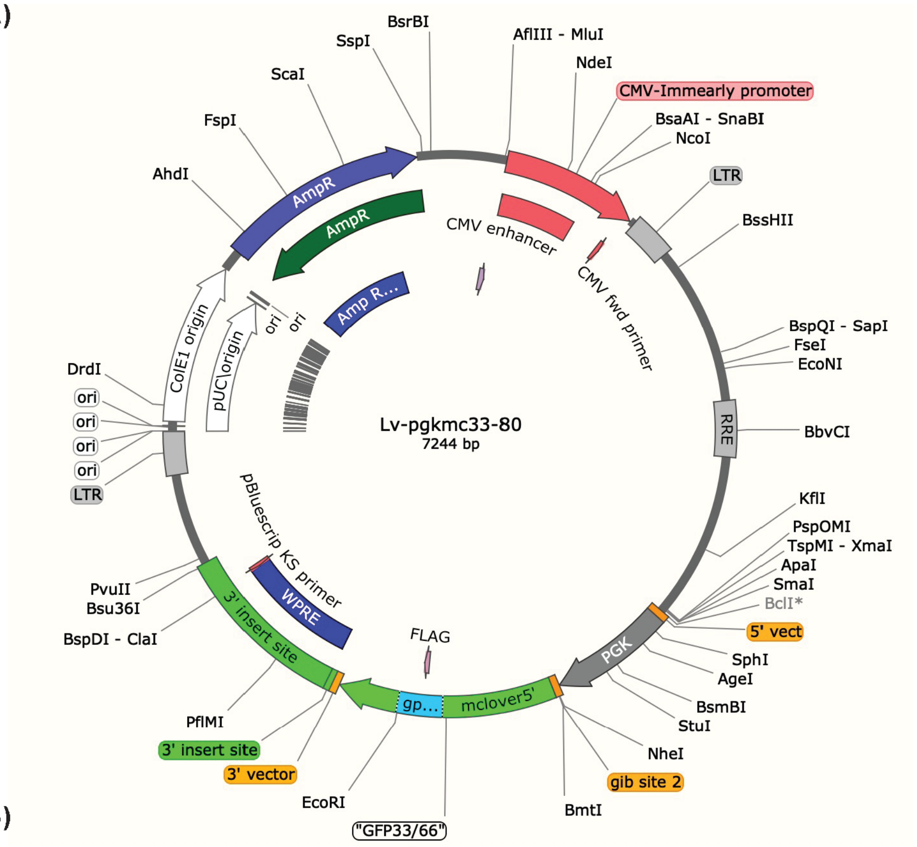

B)

GFP33/66 Sequence:

aacATGGTGAGCAAGGGCGAGGAGCTGTTCACCGGGGTGGTGCCCATCCTGGTCGAGCTG-
GACGGCGACGTAAACGGCCACAAGTTCAGCGTCCGCGGCGAGGGCGAGGGCGATGCCACCAACGGCAAGC
TGACCCTGAAGTTCATCTGCACCACCGGCAAGCTGCCCGTGCCCTGGCCCACCCTCGTGAC-
CACCTTCGGCTACGGCGTGGCCTGCTTCAGCCGCTACCCCGACCACATGAAGCAGCACGACTTCTTCAAG
TCCGCCATGCCCGAAGGCTACGTCCAGGAGCGCACCATCTCTTTCAAGGACGACGGTAC-
CTACAAGACCCGCGCCGAGGTGAAGTTCGAGGGCGACACCCTGGTGAACCGCATCGAGCTGAAGGGCATC
GACTTCAAGGAGGACGGCAACATCCTGGGGCACAAGCTGGAGTACAACTTCAACAGCCAC-
TACGTCTATATCACGGCTGGATCTGCAGGATCAGCGGCCGGCTCAGGCGAGTTTAAAGCTGTGTACAACT
TTGCAACATGCGGGATCGACTACAAGGACGACGATGACAAGGGTTTGAAC-
GGCCCTGACATTTACAAGGGAGTTTATCAGTTTAAATCCGTTGAGTTTGACGGCAGCGCGGGCAGTGCTG
CTGGGTCAGGAGAATTCGACAAGCAGAAGAACTGCATCAAGGCTAACTTCAA-
GATCCGCCACAACGTTGAGGACGGCAGCGTGCAGCTCGCCGACCACTACCAGCAGAACACCCCCATCGGC
GACGGCCCCGTGCTGCTGCCCGACAACCACTACCTGAGCCATCAGTCCAAGCTGAGCAAA-
GACCCCAACGAGAAGCGCGATCACATGGTCCTGCTGGAGTTCGTGACCGCCGCCGGGATTACACATGGCA
TGGACGAGCTGTACAAGT

Figure S6. Generation of the YUMM-GFP33/66 melanoma cell line by transduction of YUMM1.7, related to Figure S1.

A) Full sequence map of the construct used to transduce YUMM 1.7 melanoma cell lines to create YUMM-GFP33/66.

B) Confirmatory Sanger sequencing of the GP33/66 insert.

SI References

1. *Chen EY, Tan CM, Kou Y, Duan Q, Wang Z, Meirelles GV, Clark NR, Ma'ayan A. Enrichr: interactive and collaborative HTML5 gene list enrichment analysis tool. BMC Bioinformatics. 2013; 128(14).*
2. Street, K., Risso, D., Fletcher, R.B., Das, D., Ngai, J., Yosef, N., Purdom, E. and Dudoit, S., 2018. Slingshot: cell lineage and pseudotime inference for single-cell transcriptomics. BMC genomics, 19(1), pp.1-16
3. Wong, Pok Fai, et al. "Multiplex Quantitative Analysis of Tumor-Infiltrating Lymphocytes and Immunotherapy Outcome in Metastatic MelanomaTIL Profiles and Melanoma Anti–PD-1 Response." *Clinical Cancer Research* 25.8 (2019): 2442-2449.
4. Chen EY, Tan CM, Kou Y, Duan Q, Wang Z, Meirelles GV, Clark NR, Ma'ayan A.
5. Enrichr: interactive and collaborative HTML5 gene list enrichment analysis tool. BMC Bioinformatics. 2013; 128(14).
6. Kuleshov MV, Jones MR, Rouillard AD, Fernandez NF, Duan Q, Wang Z, Koplev S, Jenkins SL, Jagodnik KM, Lachmann A, McDermott MG, Monteiro CD, Gundersen GW, Ma'ayan A.
7. Enrichr: a comprehensive gene set enrichment analysis web server 2016 update. Nucleic Acids Research. 2016; gkw377 .
8. Xie Z, Bailey A, Kuleshov MV, Clarke DJB., Evangelista JE, Jenkins SL, Lachmann A, Wojciechowicz ML, Kropiwnicki E, Jagodnik KM, Jeon M, & Ma’ayan A.
9. Gene set knowledge discovery with Enrichr. Current Protocols, 1, e90. 2021. doi: 10.1002/cpz1.90
10. Meeth, K., Wang, J. X., Micevic, G., Damsky, W., & Bosenberg, M. W. (2016). The YUMM lines: a series of congenic mouse melanoma cell lines with defined genetic alterations. *Pigment cell & melanoma research*, *29*(5), 590-597.
